# Supplementary material for: Synthesis and Structure of the Bis- and Tris-Polyhedral Hybrid Carboranoclathrochelates with Functionalizing Biorelevant Substituents—The Derivatives of Propargylamine Iron(II) Clathrochelates with Terminal Triple C≡C Bond(s)
Source: Molecules. 2021 Jun 14;26(12):3635. doi: 10.3390/molecules26123635 (PMC8232327; doi:10.3390/molecules26123635)
Supplement: Supplementary file 1 [file molecules-26-03635-s001.zip › molecules-1248311-supplementary.pdf]

Supplementary Materials

for

Synthesis and Structure of the Bis- and Tris-Polyhedral Hybrid Carboranoclathrochelates with Functionalizing Biorelevant Substituents—the Derivatives of Propargylamine Iron(II) Clathrochelates with Terminal Triple C≡C Bond(s)

Genrikh E. Zelinskii,[1,2] Ilya P. Limarev,[1,2] Anna V. Vologzhanina,[2] Valentina A. Olshevskaya,[2] Anton V. Makarenkov,[2] Pavel V. Dorovatovskii,[3] Alexander S. Chuprin,[2] Mikhail A. Vershinin,[4] Semyon V. Dudkin[2] and Yan Z. Voloshin[1,2*]

[1]*Kurnakov Institute of General and Inorganic Chemistry*

of the Russian Academy of Sciences, 31 Leninsky prosp., 119991 Moscow, Russia

[2]*Nesmeyanov Institute of Organoelement Compounds*

of the Russian Academy of Sciences, 28 Vavilova st., 119991 Moscow, Russia

[3]*National Research Center Kurchatov Institute,*

1 Kurchatova pl., 123098 Moscow, Russia

[4]*Nikolaev Institute of Inorganic Chemistry*

of the Siberian Branch of the Russian Academy of Sciences,

3 Lavrentieva prosp., 630090 Novosibirsk, Russia

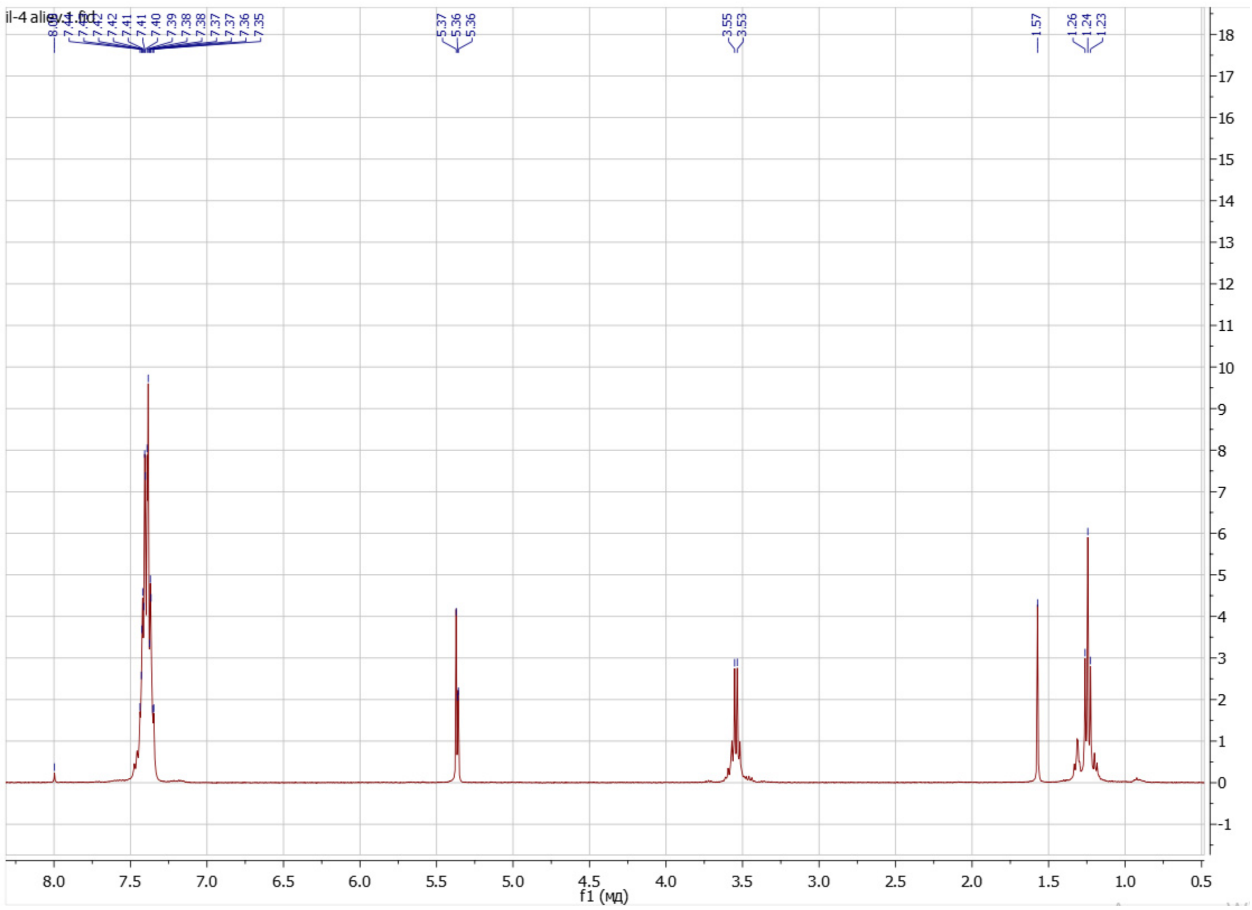

Figure S1. ^{1}H NMR spectrum of the clathrochelate precursor $FeBd_2(ClGmDea)(BF)_2$ (400.13 MHz, CD_2Cl_2, 20°C).

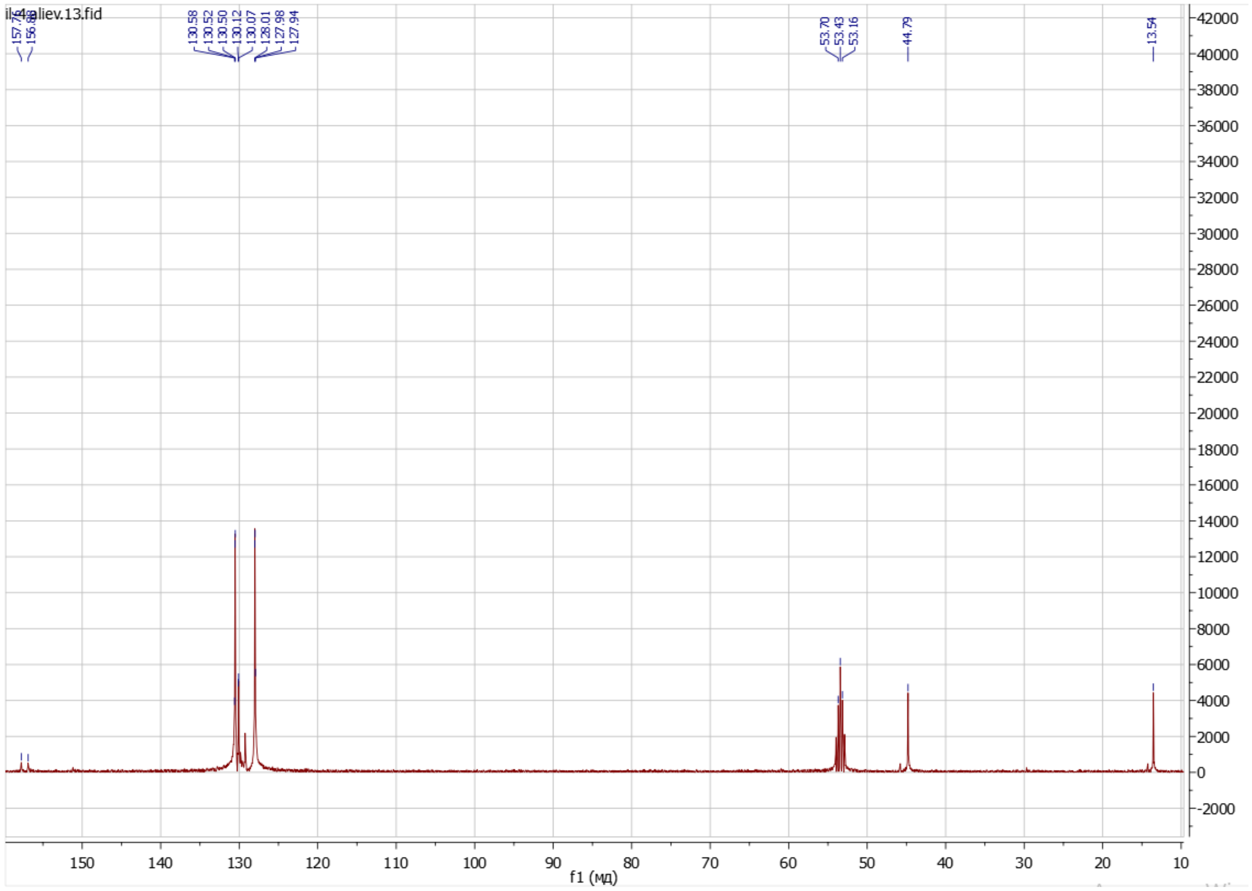

Figure S2. $^{13}C\{^1H\}$ NMR spectrum of the clathrochelate precursor $FeBd_2(ClGmDea)(BF)_2$ (400.13 MHz, CD_2Cl_2, 20°C).

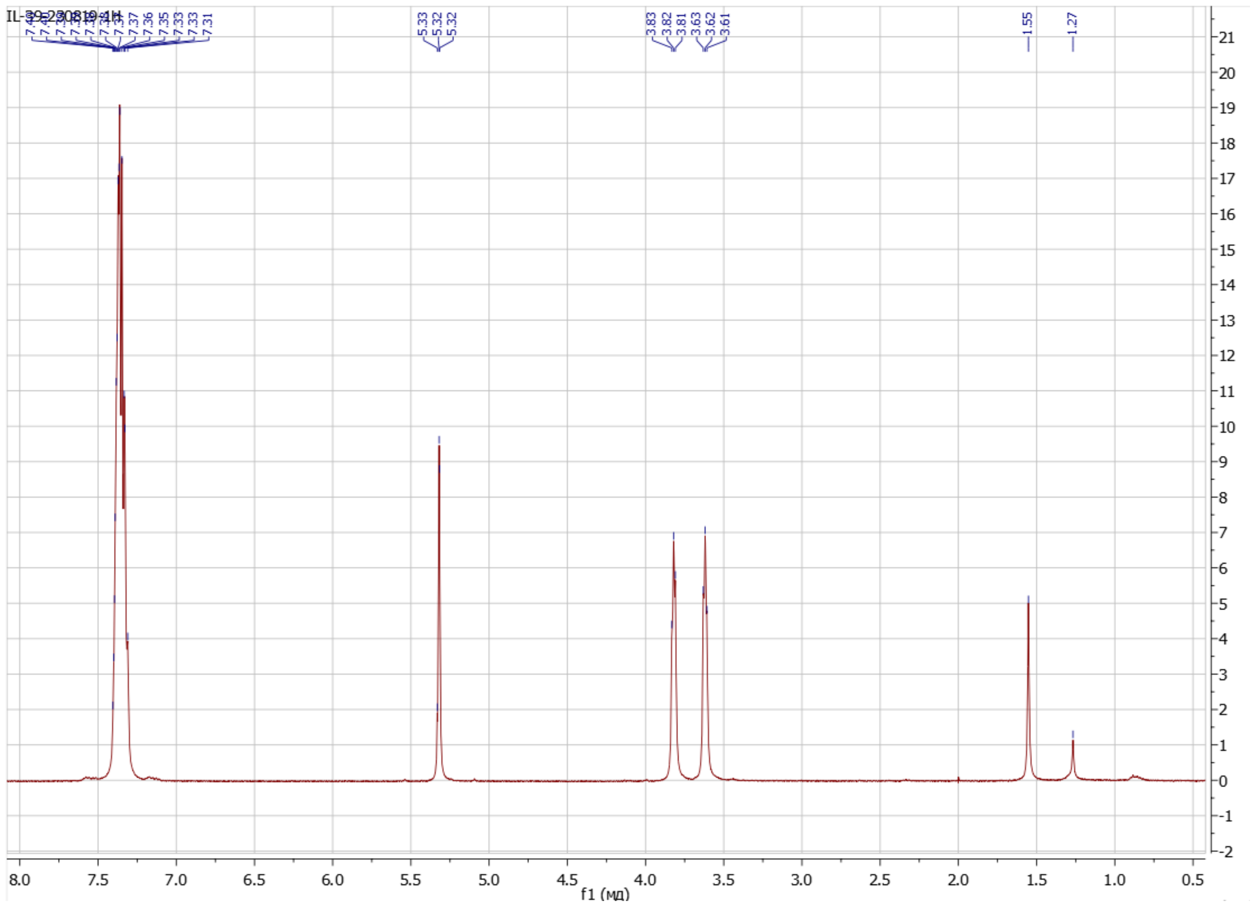

Figure S3. ^{1}H NMR spectrum of the clathrochelate precursor $FeBd_2(ClGmMorph)(BF)_2$ (400.13 MHz, CD_2Cl_2, 20°C).

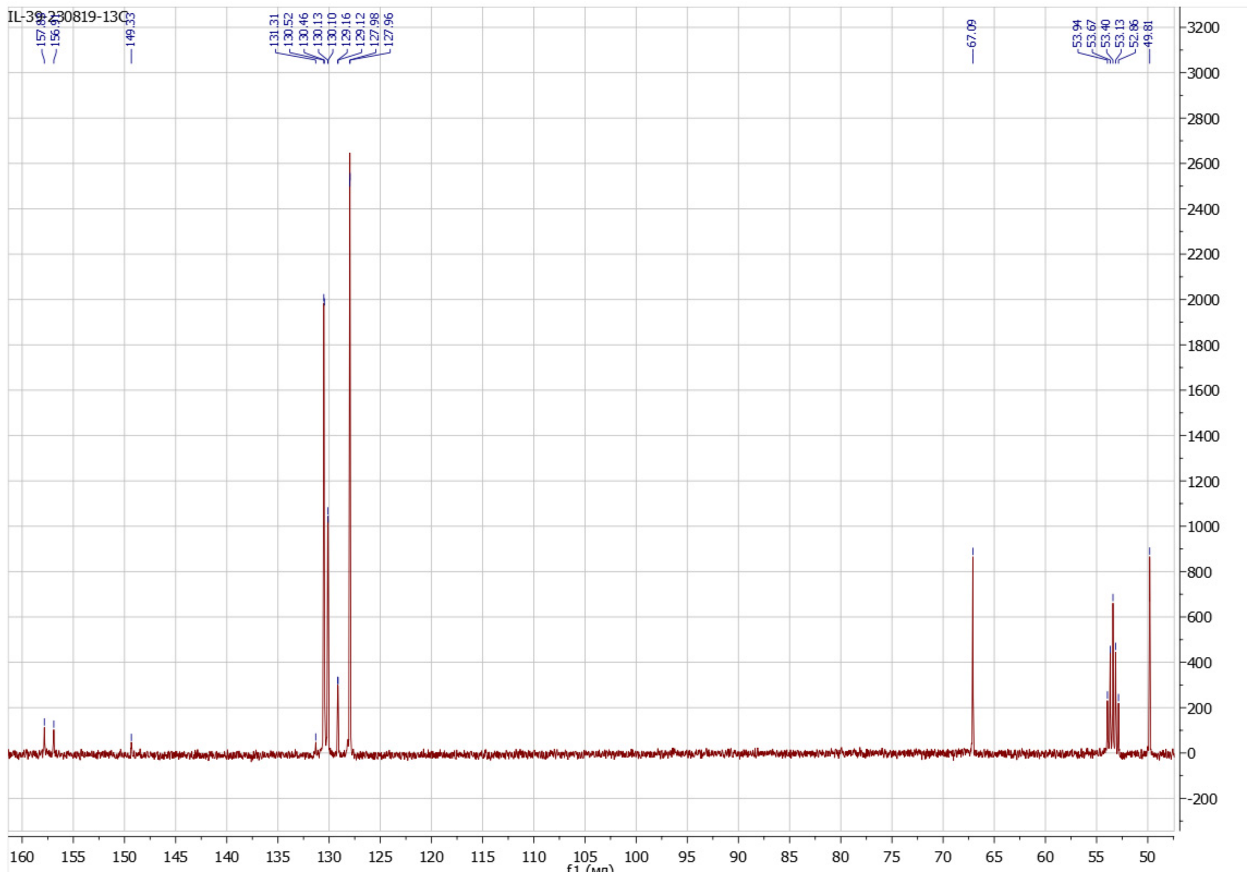

Figure S4. $^{13}C\{^1H\}$ NMR spectrum of the clathrochelate precursor $FeBd_2(ClGmMorph)(BF)_2$ (400.13 MHz, CD_2Cl_2, 20°C).

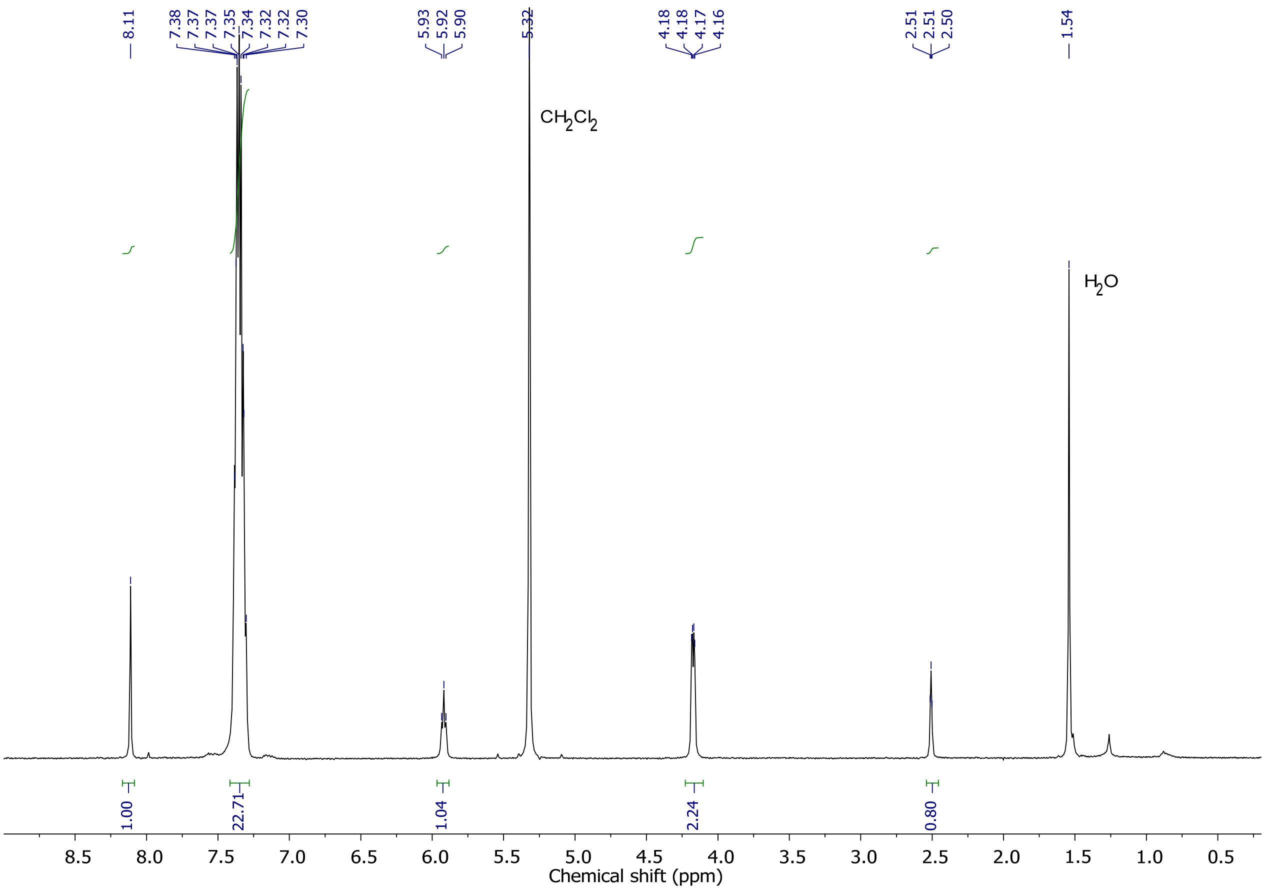

Figure S5. ^{1}H NMR spectrum of the clathrochelate $FeBd_2(HGmProp)(BF)_2$ (400.13 MHz, CD_2Cl_2, 20°C).

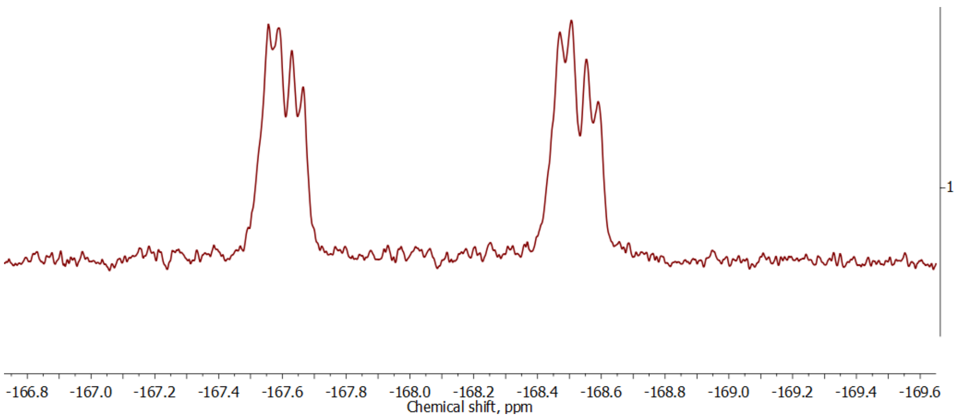

Figure S6. $^{19}F\{^{1}H\}$ NMR spectrum of the carboranoclathrochelate complex $FeBd_2(HGmProp)(BF)_2$

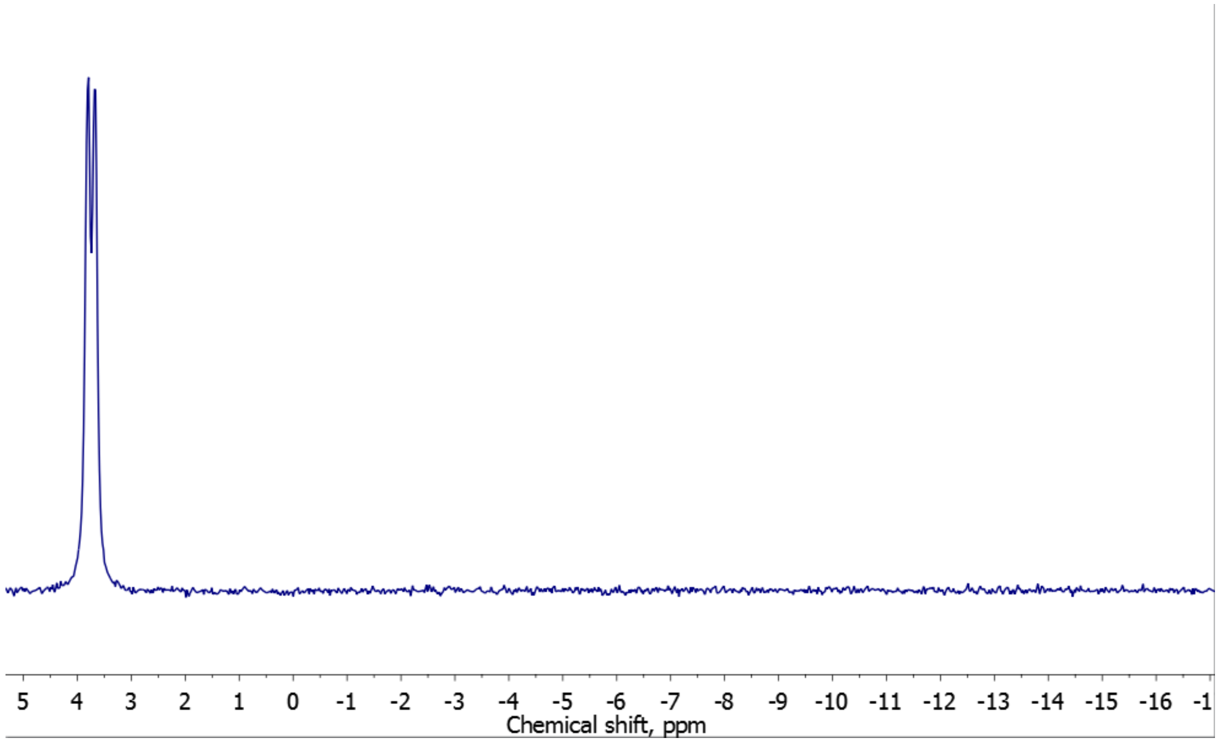

Figure S7. $^{11}B\{^1H\}$ NMR spectrum of the clathrochelate $FeBd_2(HGmProp)(BF)_2$.

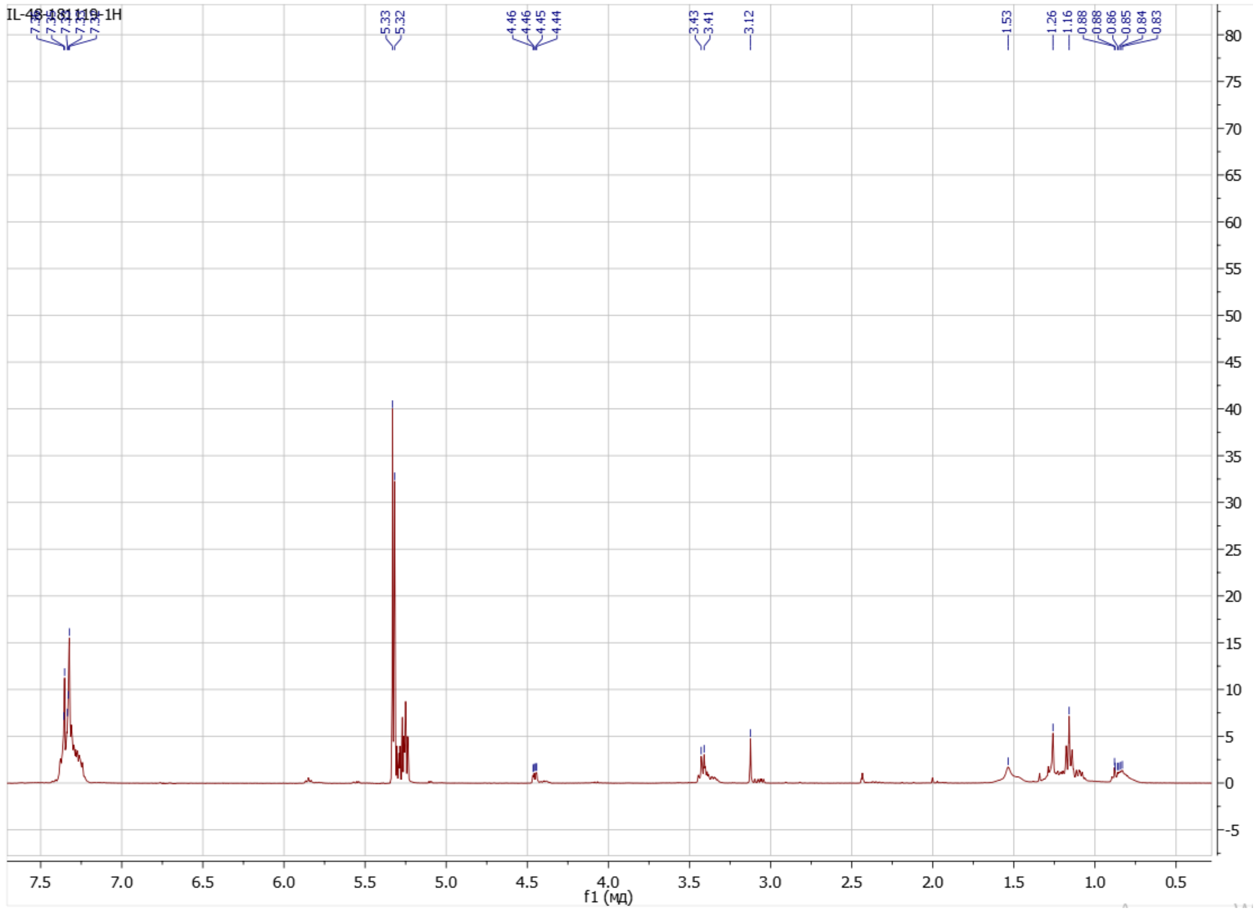

Figure S8.^{1}H NMR spectrum of the complex $FeBd_2(PropGmDea)(BF)_2$ (400.13 MHz, CD_2Cl_2, 20°C).

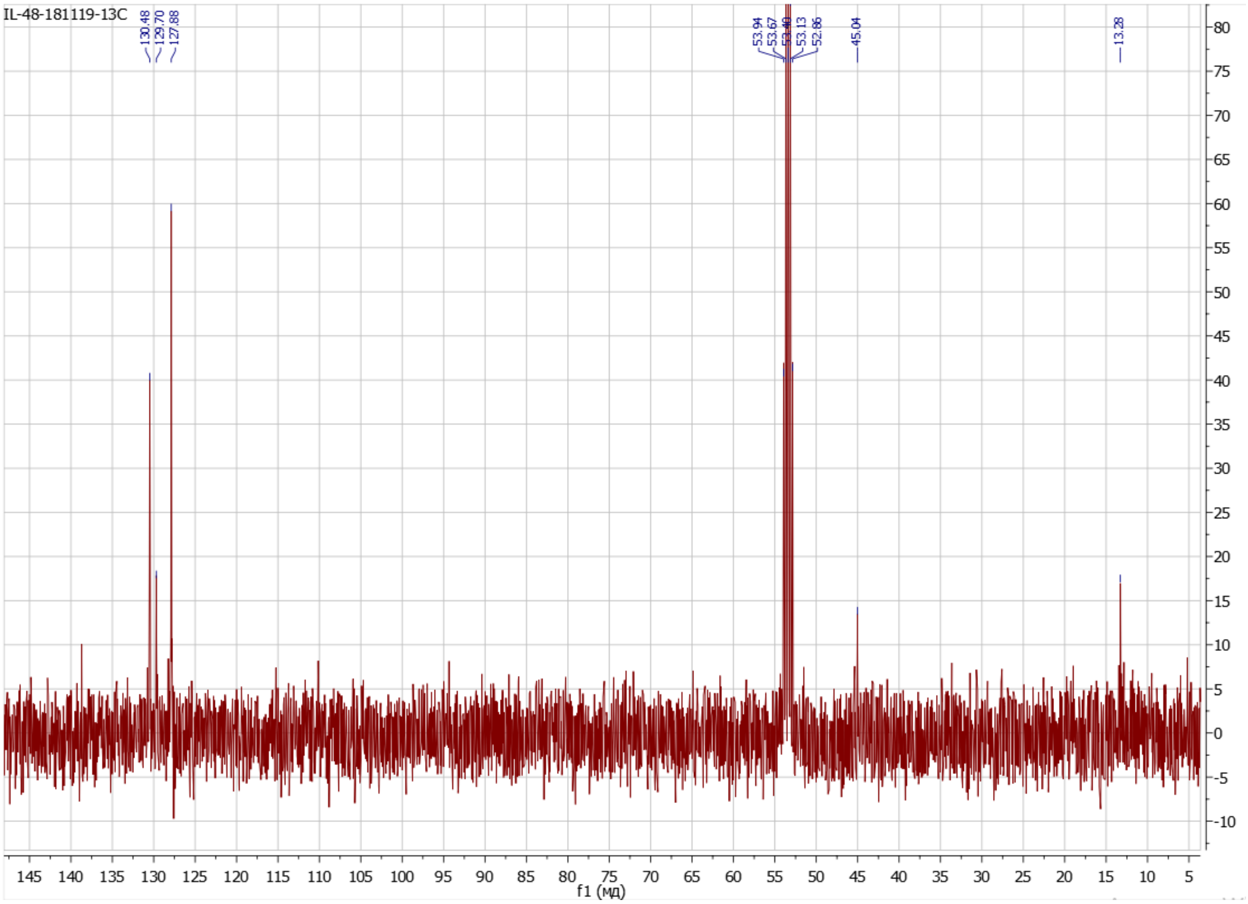

Figure S9. $^{13}C\{^{1}H\}$ NMR spectrum of the complex $FeBd_2(PropGmDea)(BF)_2$ (400.13 MHz, CD_2Cl_2, 20°C).

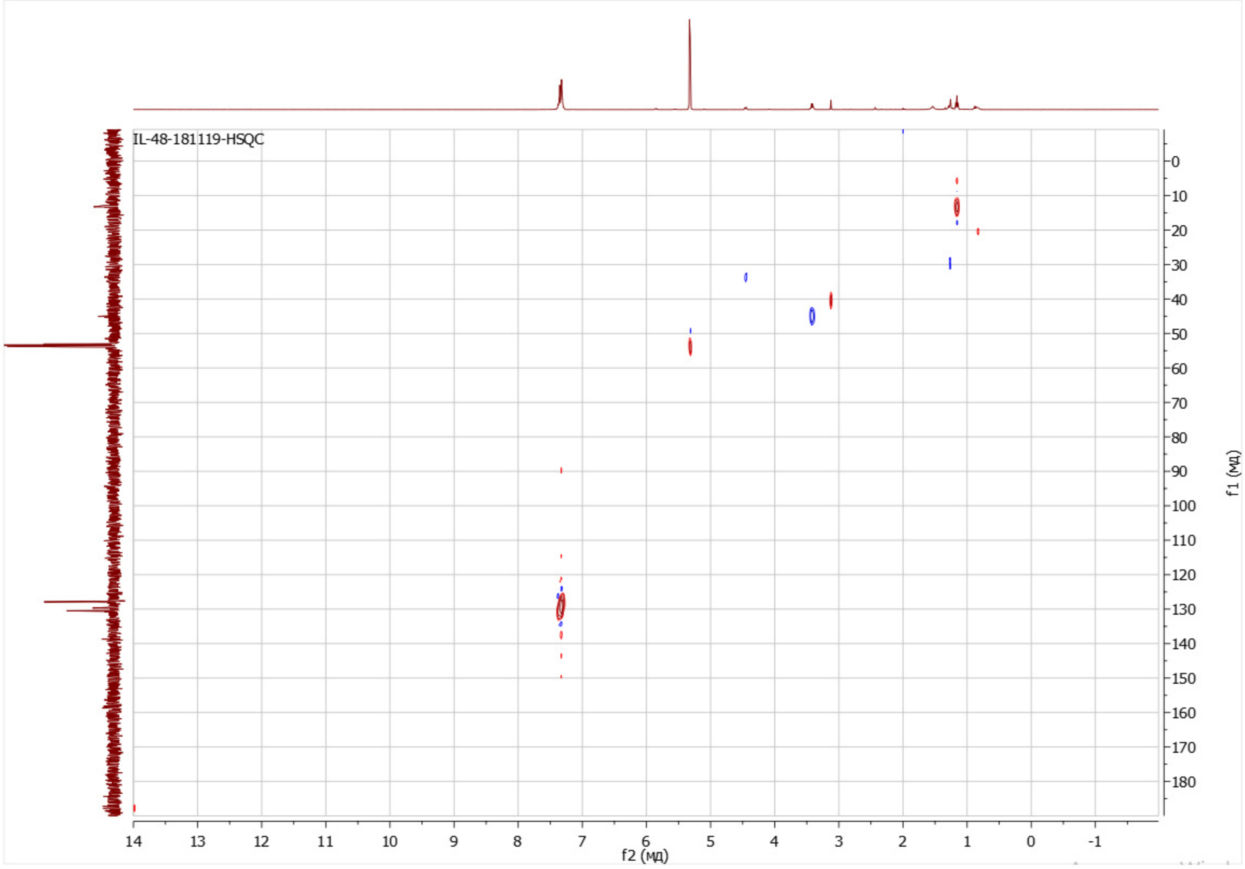

Figure S10. 2D HSQC NMR spectrum of the complex $FeBd_2(PropGmDea)(BF)_2$ (400.13 MHz, CD_2Cl_2, 20°C).

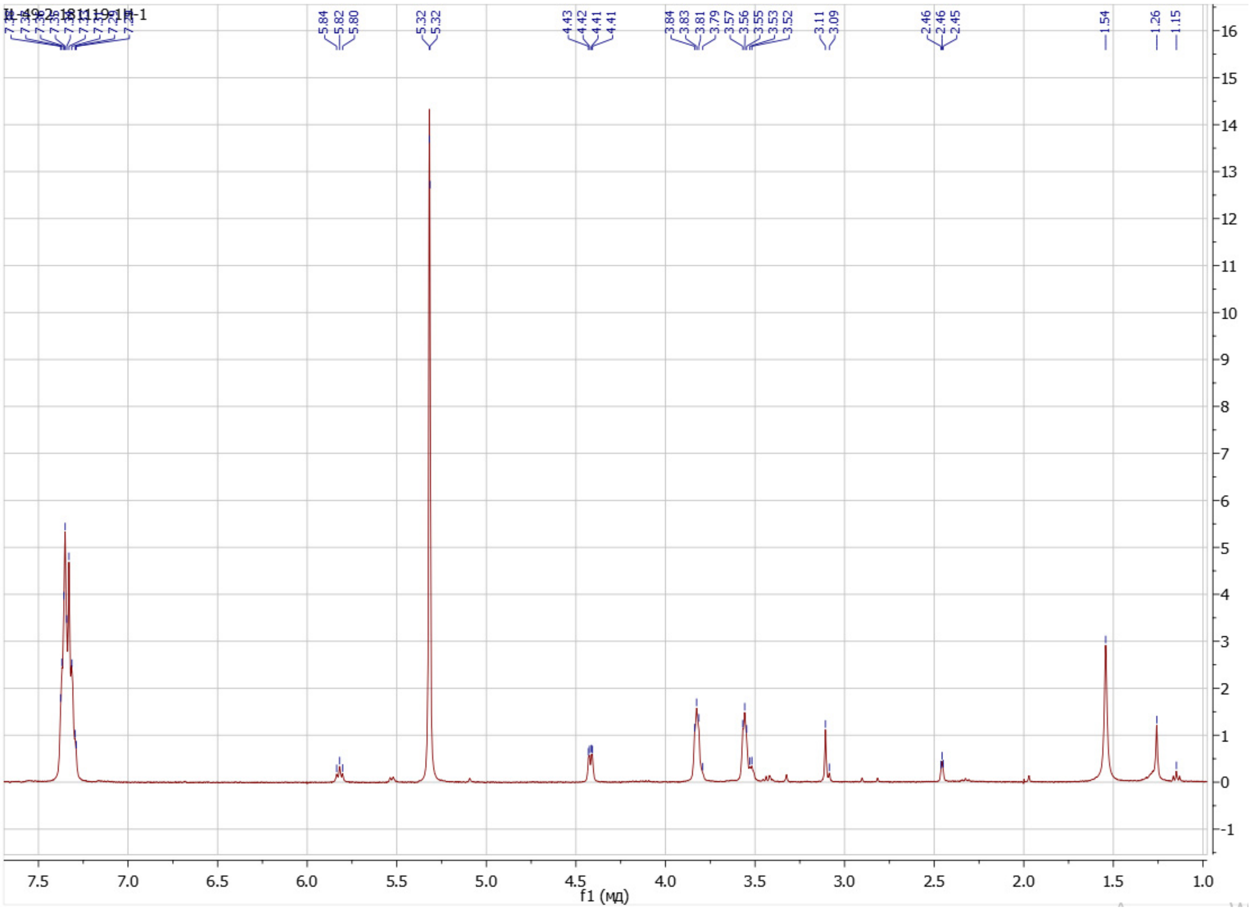

Figure S11. ^{1}H NMR spectrum of the complex $FeBd_2(PropGmMorph)(BF)_2$ (400.13 MHz, CD_2Cl_2, 20°C).

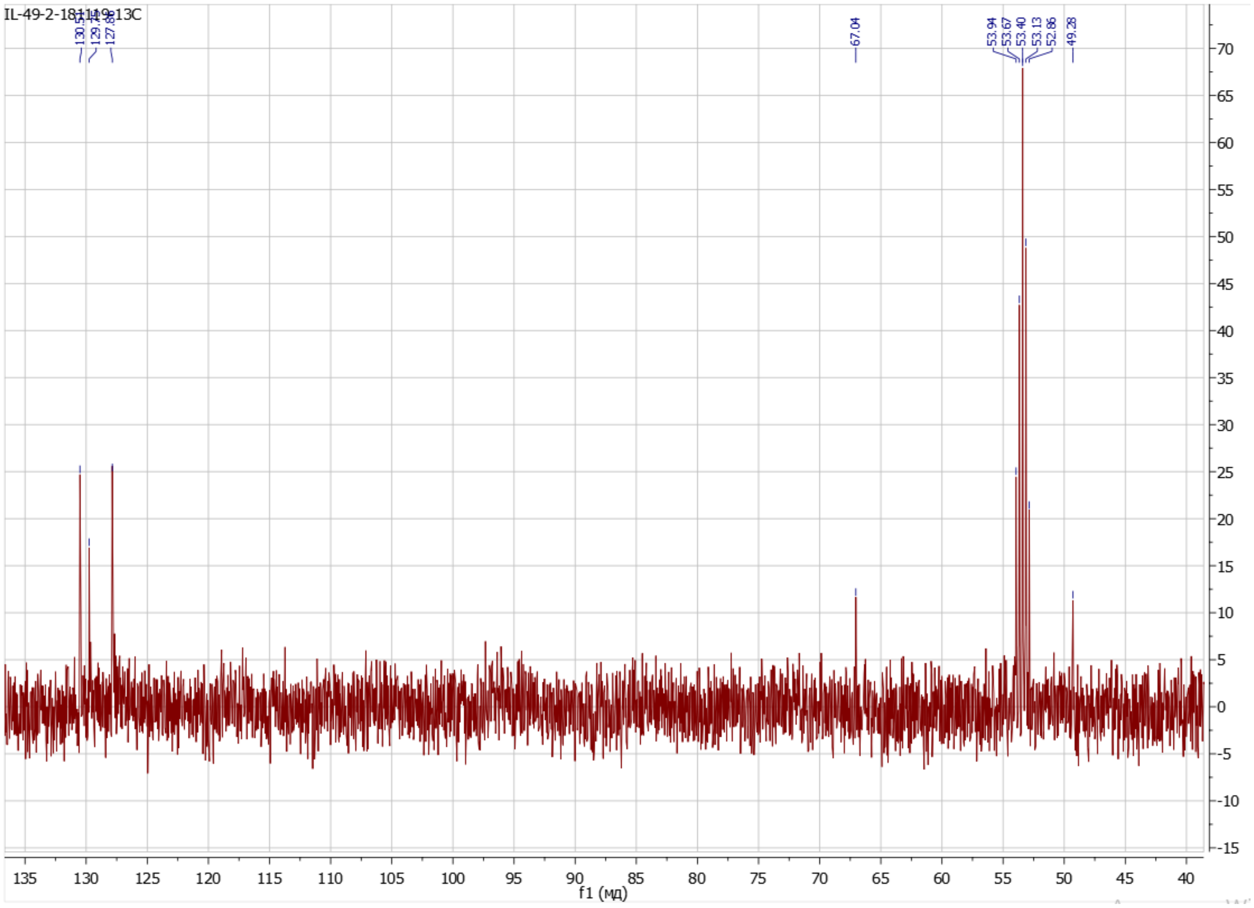

Figure S12. $^{13}C\{^1H\}$ NMR spectrum of the complex $FeBd_2(PropGmMorph)(BF)_2$ (400.13 MHz, CD_2Cl_2, 20°C).

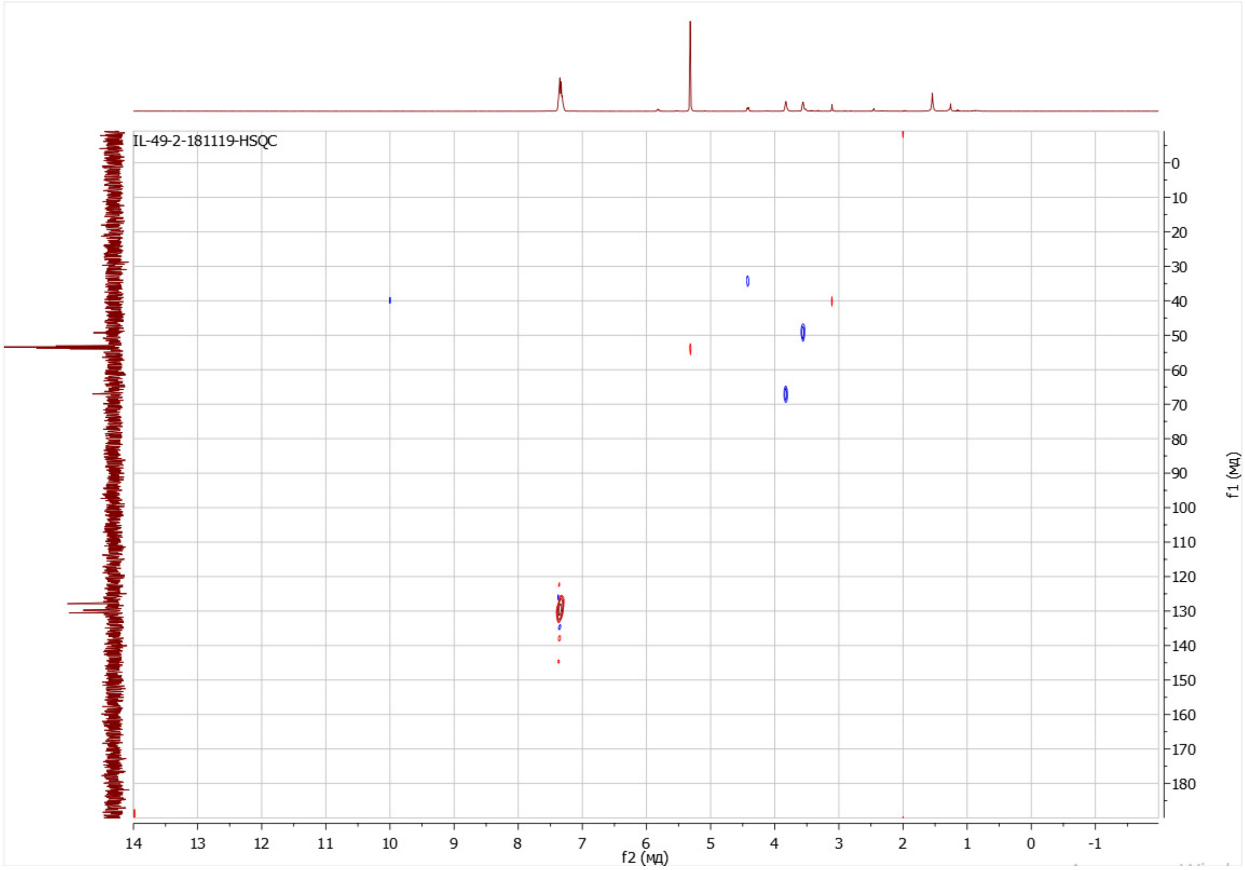

Figure S13. 2D HSQC NMR spectrum of the complex $FeBd_2(PropGmMorph)(BF)_2$ (400.13 MHz, CD_2Cl_2, 20°C).

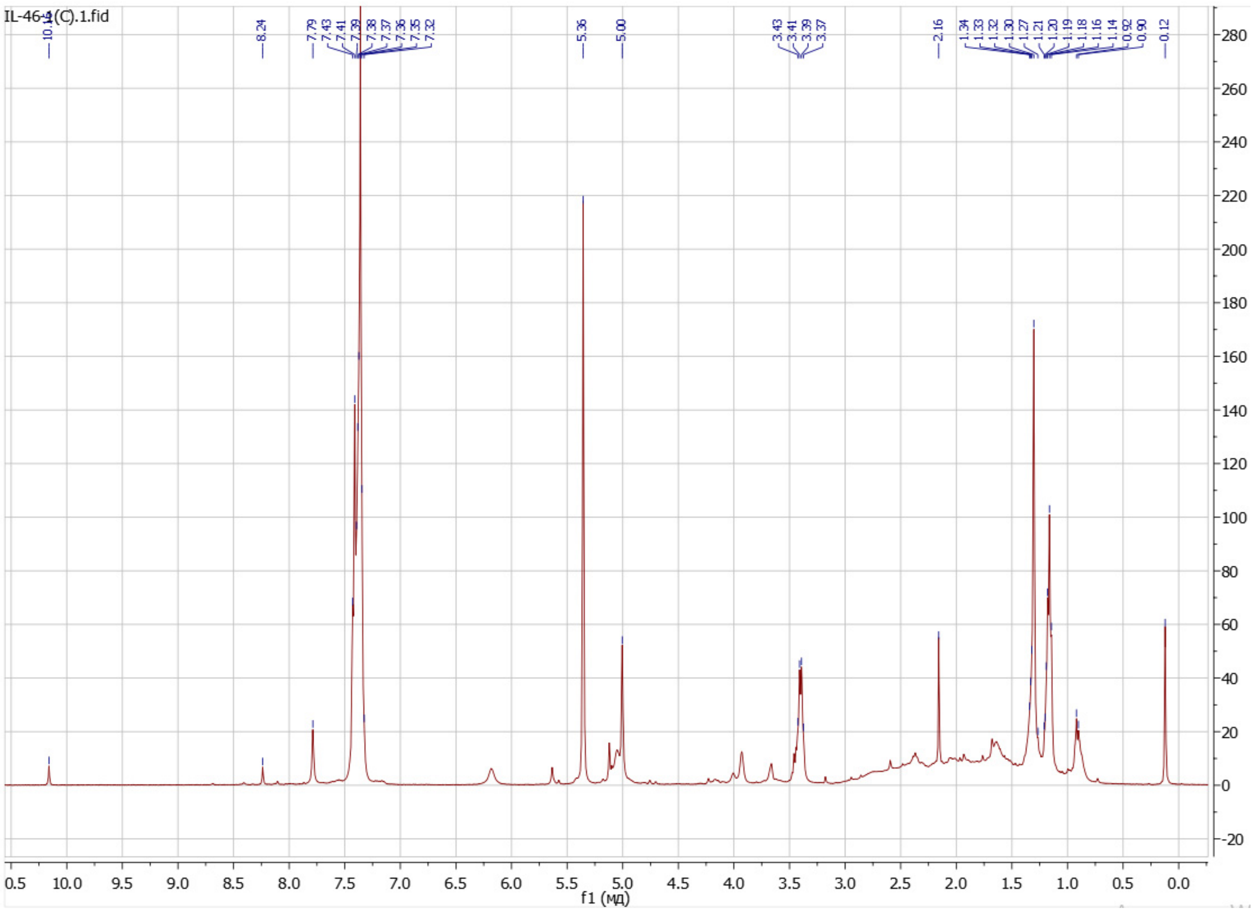

Figure S14. ^{1}H NMR spectrum of the carboranoclathrochelate $FeBd_2(DeaGmSpCarb)(BF)_2$ (400.13 MHz, CD_2Cl_2, 20°C).

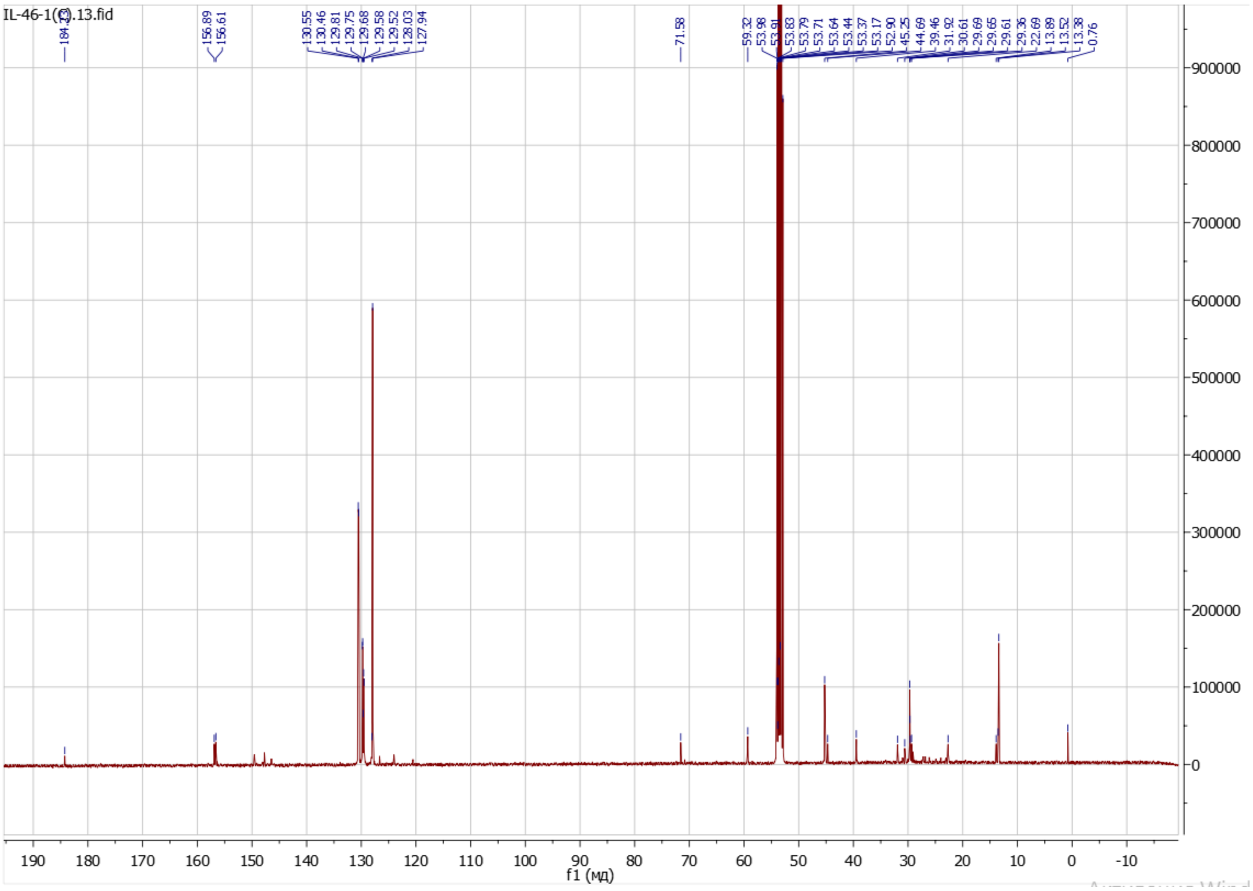

Figure S15. $^{13}C\{^{1}H\}$ NMR spectrum of the carboranoclathrochelate $FeBd_2(DeaGmSpCarb)(BF)_2$ (400.13 MHz, CD_2Cl_2, 20°C).

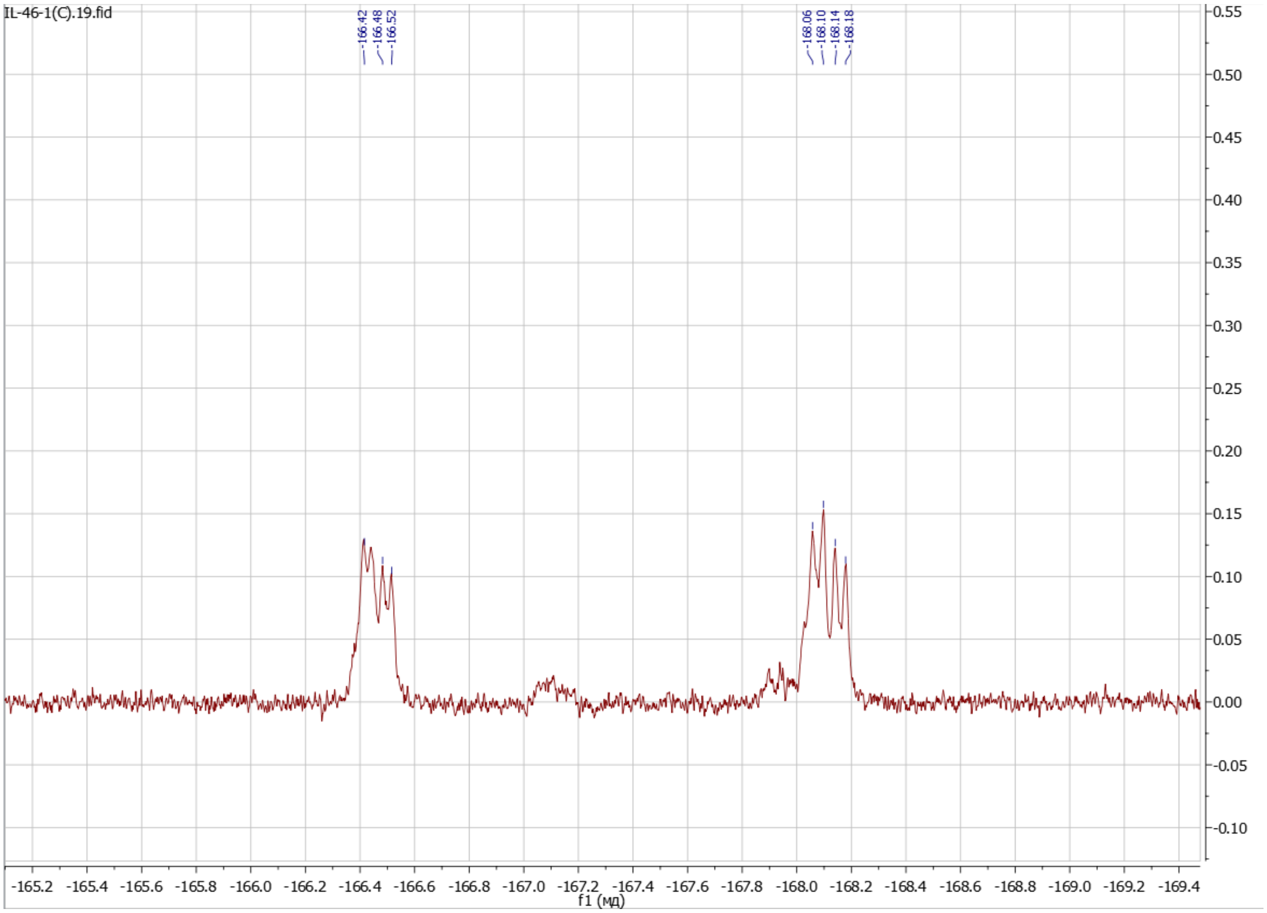

δ19F

Figure S16. Fragment of $^{19}F\{^{1}H\}$ NMR spectrum of the carboranoclathrochelate $FeBd_2(DeaGmSpCarb)(BF)_2$ (400.13 MHz, CD_2Cl_2, 20°C).

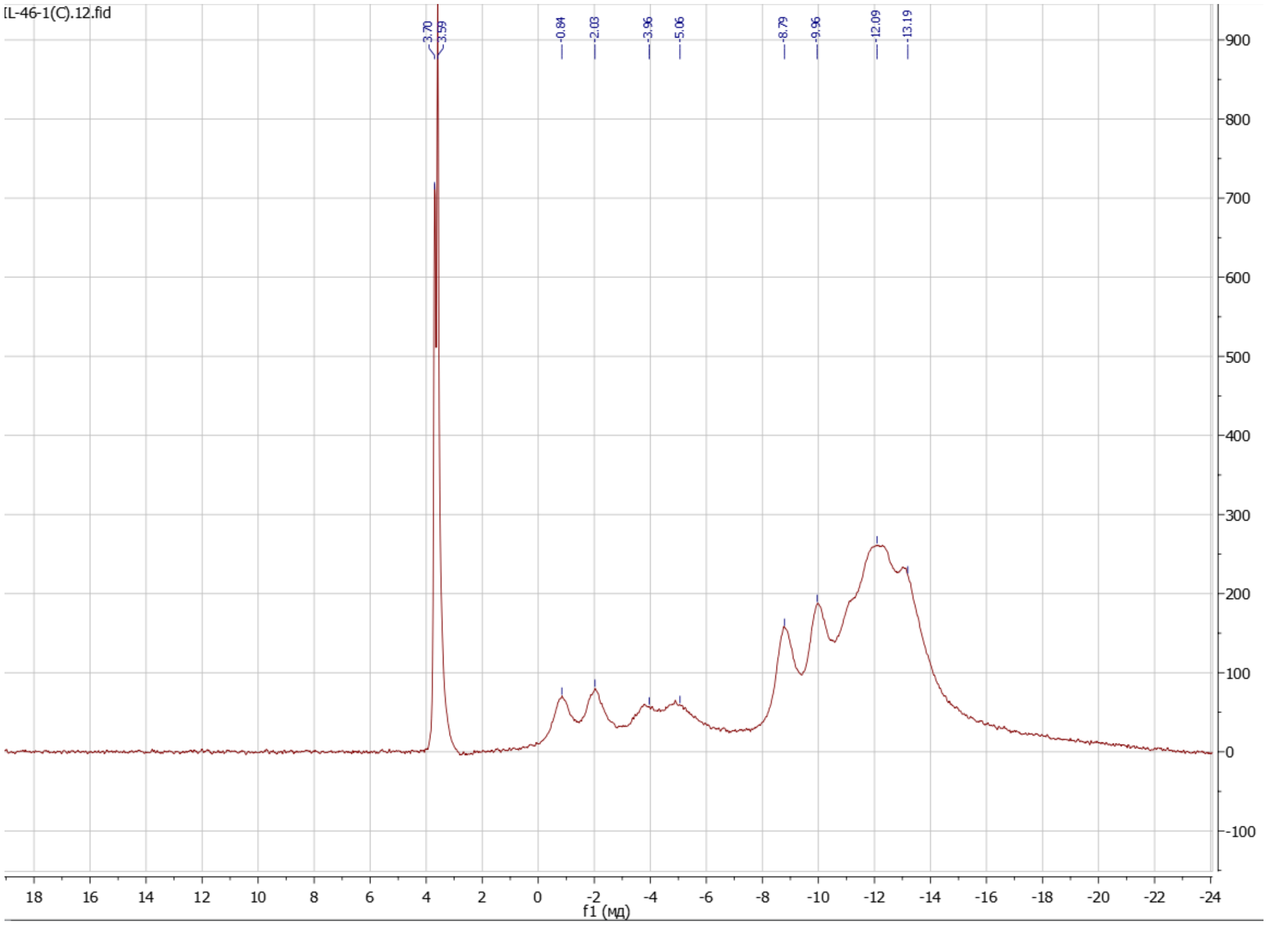

Figure S17. Fragment of the ${}^{11}B\{{}^{1}H\}$ NMR spectrum of the carboranoclathrochelate $FeBd_2(DeaGmSpCarb)(BF)_2$ (400.13 MHz, CD_2Cl_2, 20°C).

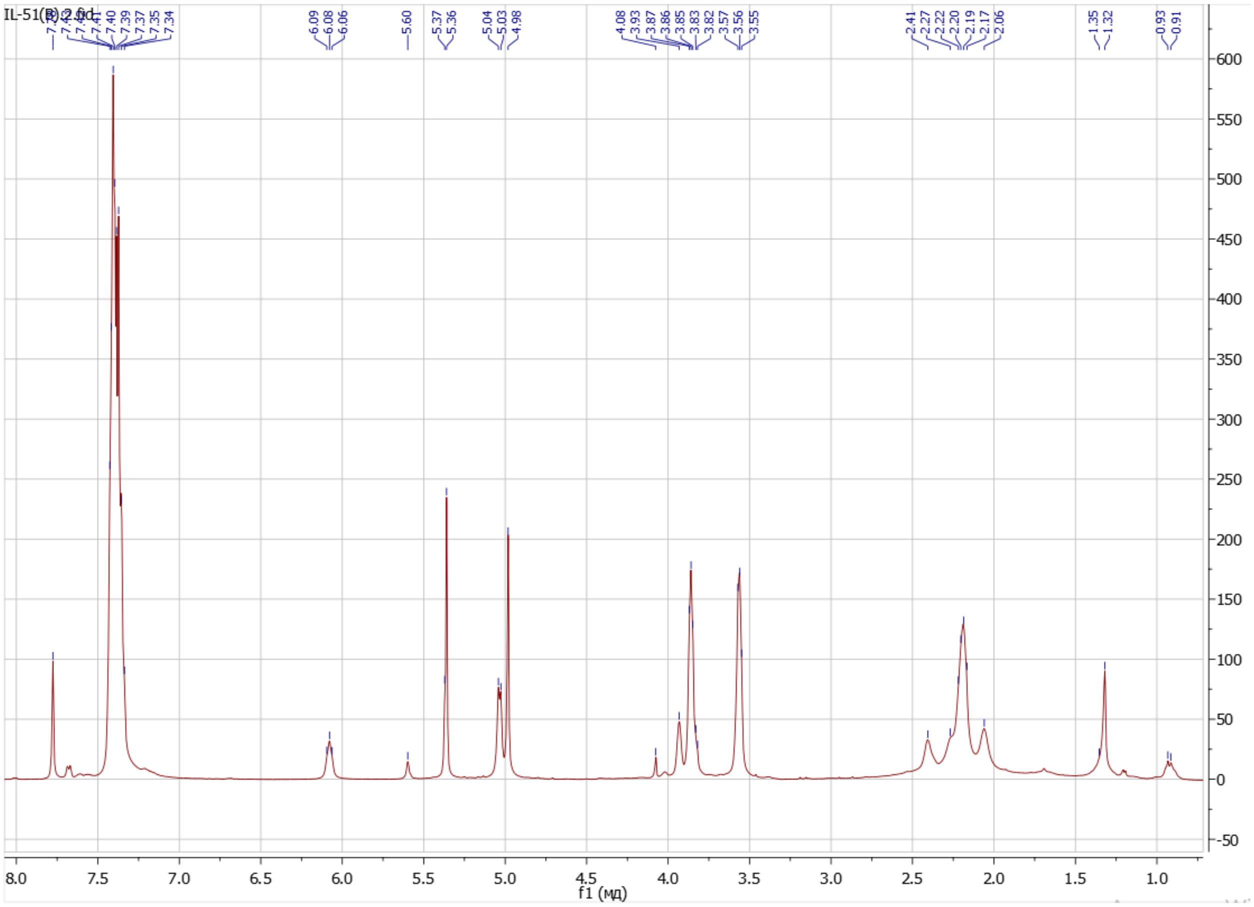

Figure S18. ^{1}H NMR spectrum of the carboranoclathrochelate $FeBd_2(MorphGmSpCarb)(BF)_2$ (400.13 MHz, CD_2Cl_2, 20°C).

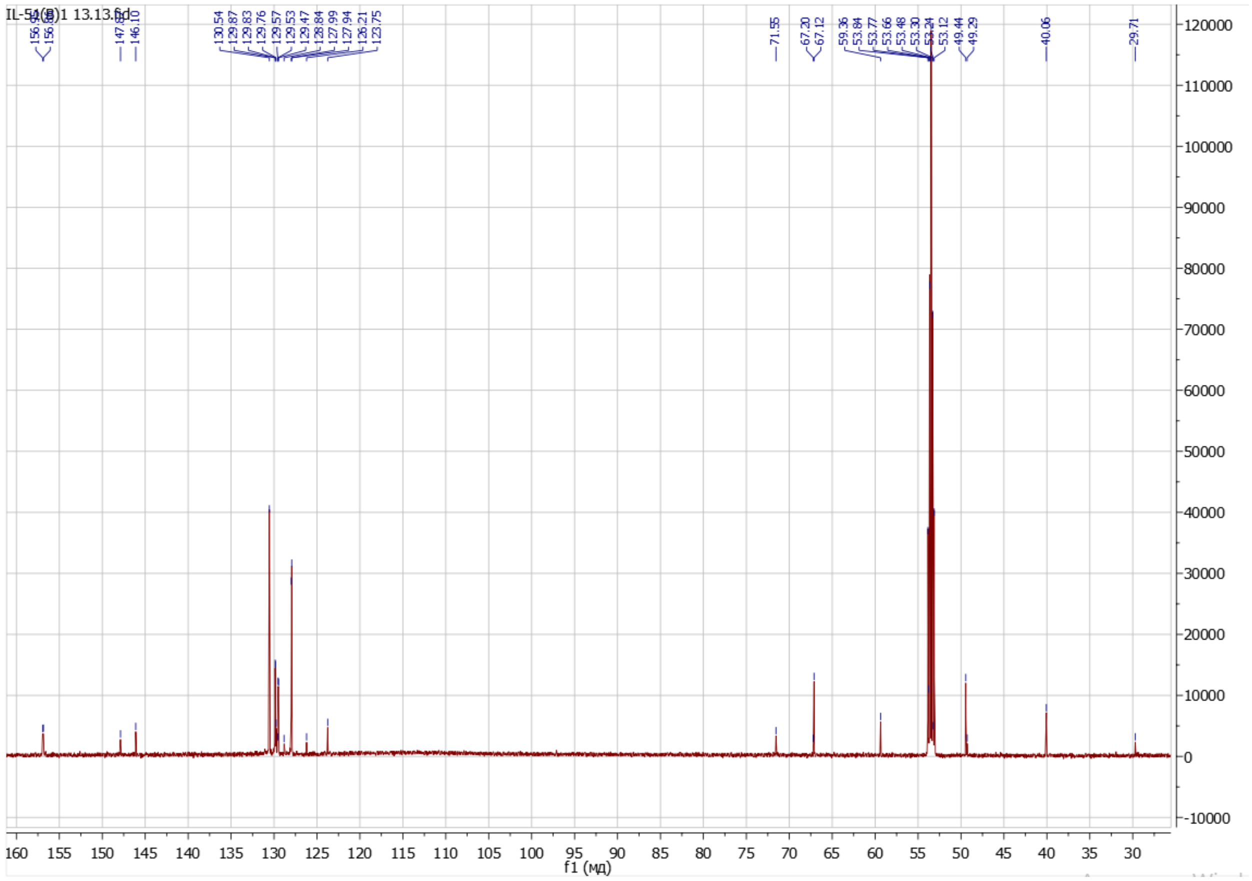

Figure S19. $^{13}C\{^{1}H\}$ NMR spectrum of the carboranoclathrochelate $FeBd_2(MorphGmSpCarb)(BF)_2$ (400.13 MHz, CD_2Cl_2, 20°C).

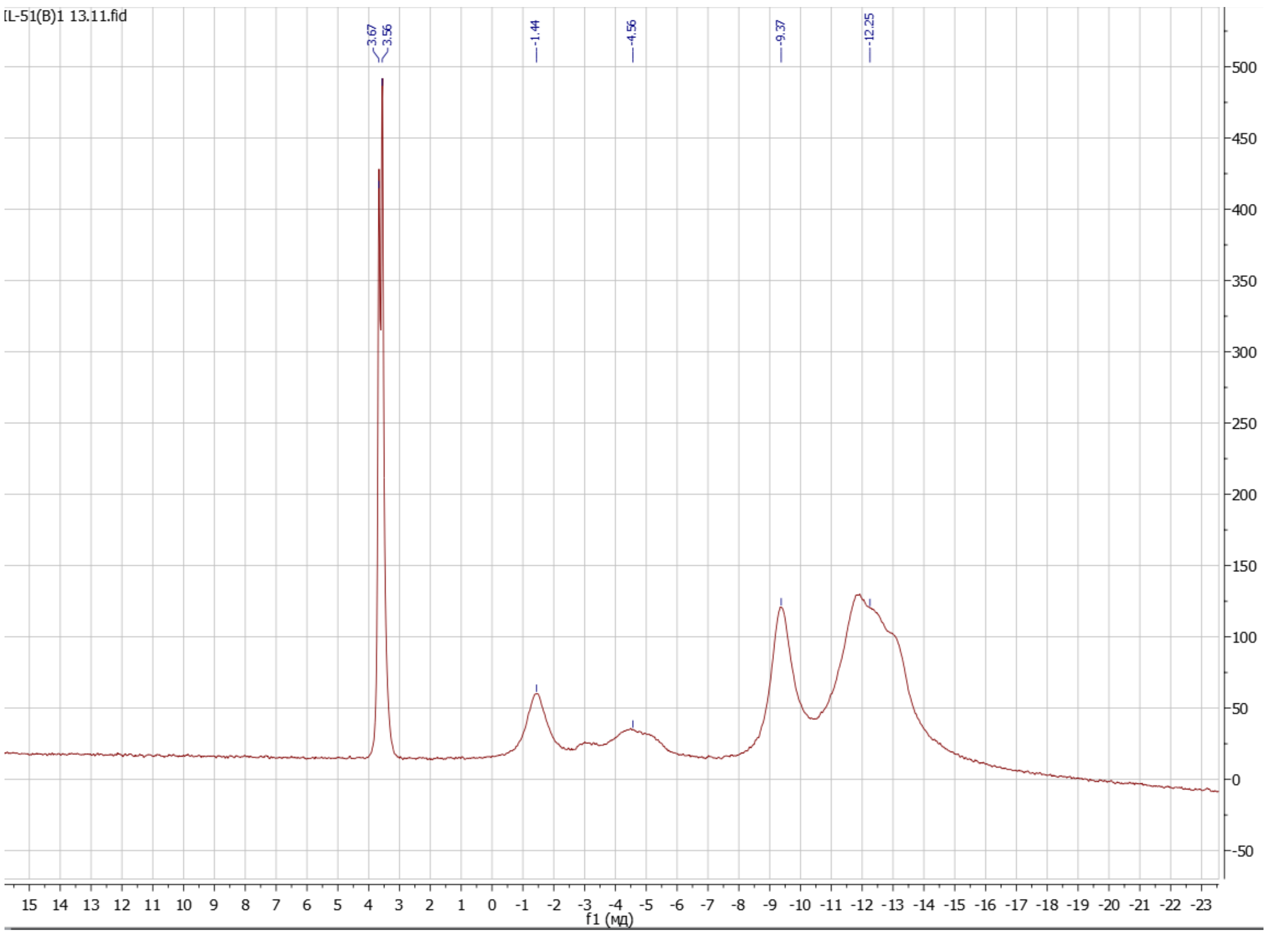

Figure S20. Fragment of ^{11}B NMR spectrum of the carboranoclathrochelate $FeBd_2(MorphGmSpCarb)(BF)_2$ (400.13 MHz, CD_2Cl_2, 20°C).

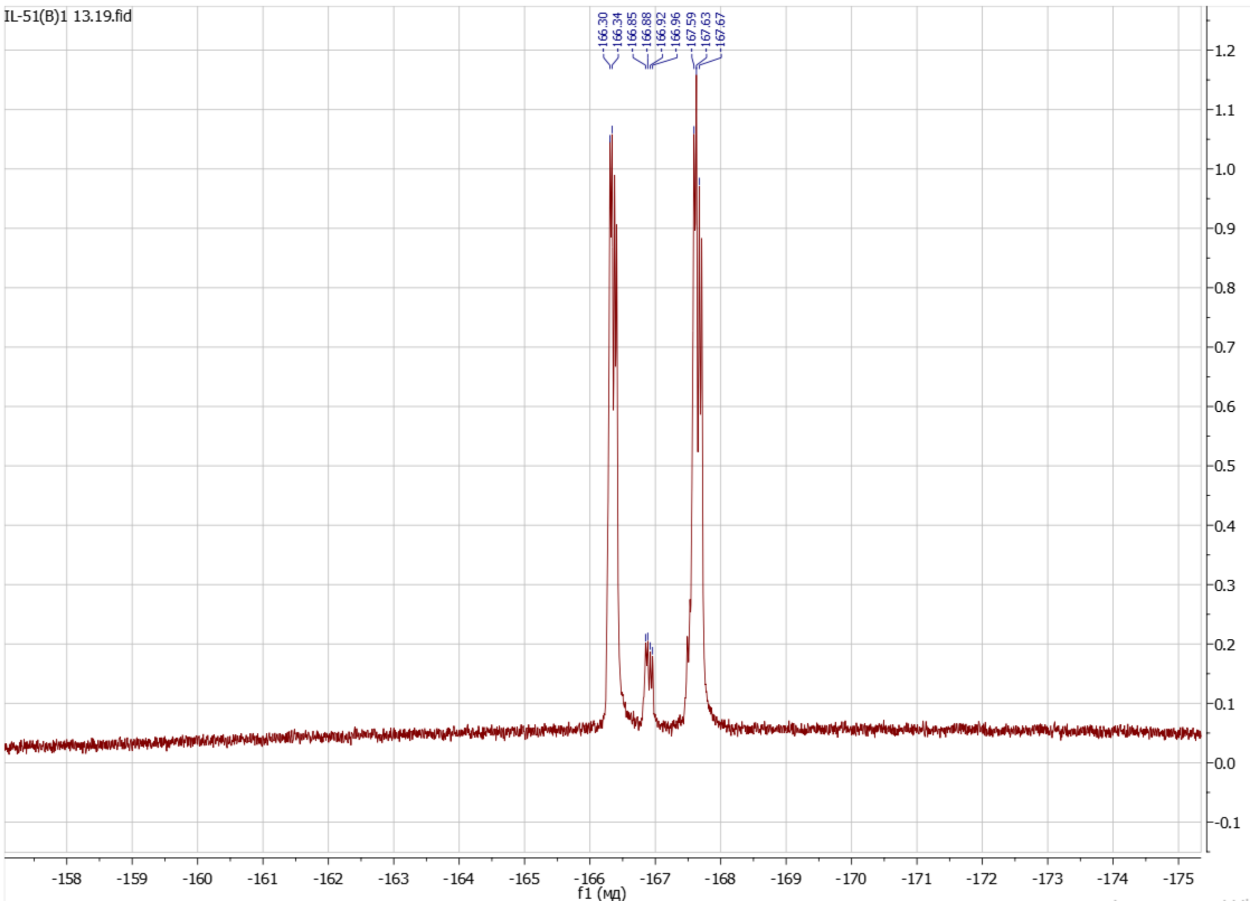

Figure S21. ^{19}F NMR spectrum of the carboranoclathrochelate $FeBd_2(MorphGmSpCarb)(BF)_2$ (400.13 MHz, CD_2Cl_2, 20°C).

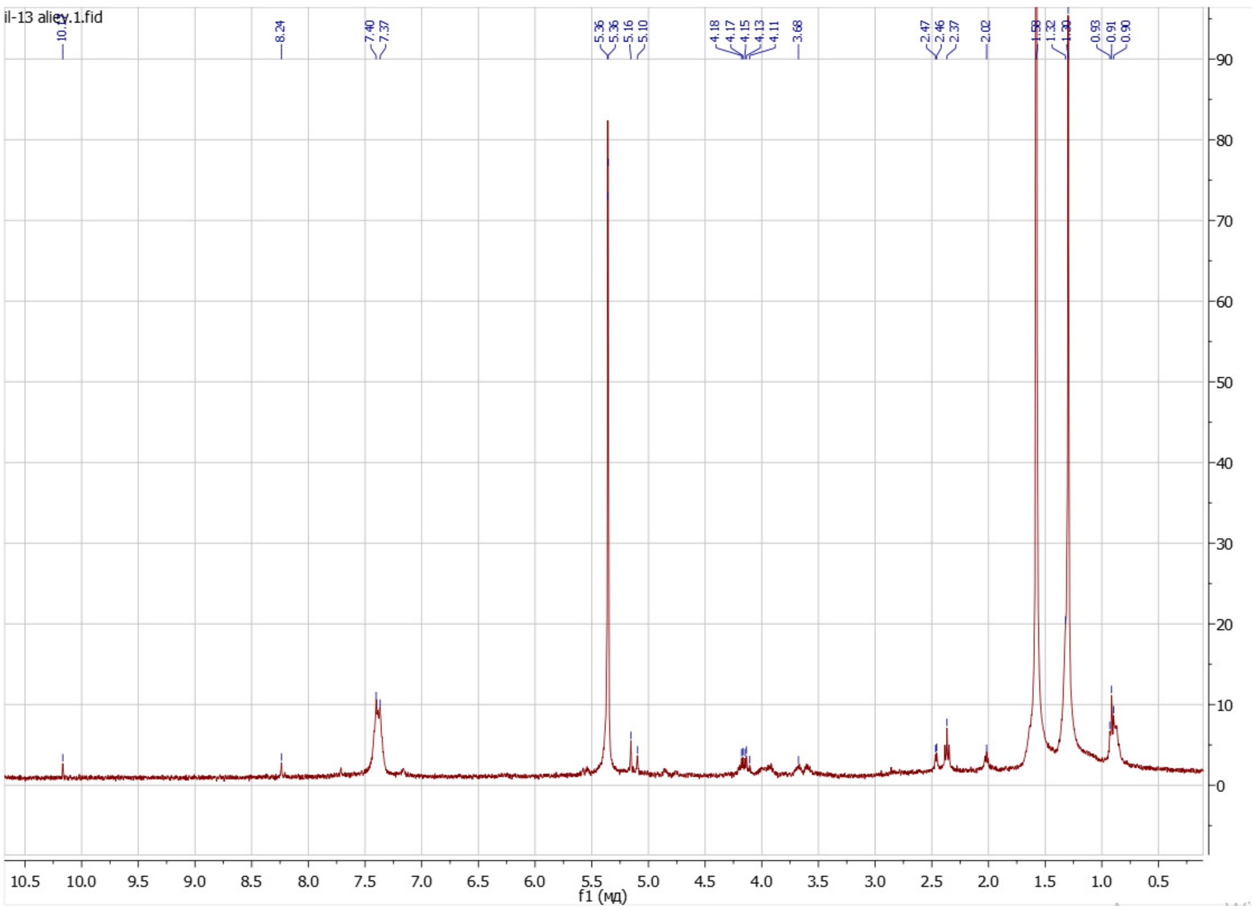

Figure S22. ^{1}H NMR spectrum of the carboranoclathrochelate $FeBd_2(Gm(SpCarb)_2)(BF)_2$ (400.13 MHz, CD_2Cl_2, 20°C).

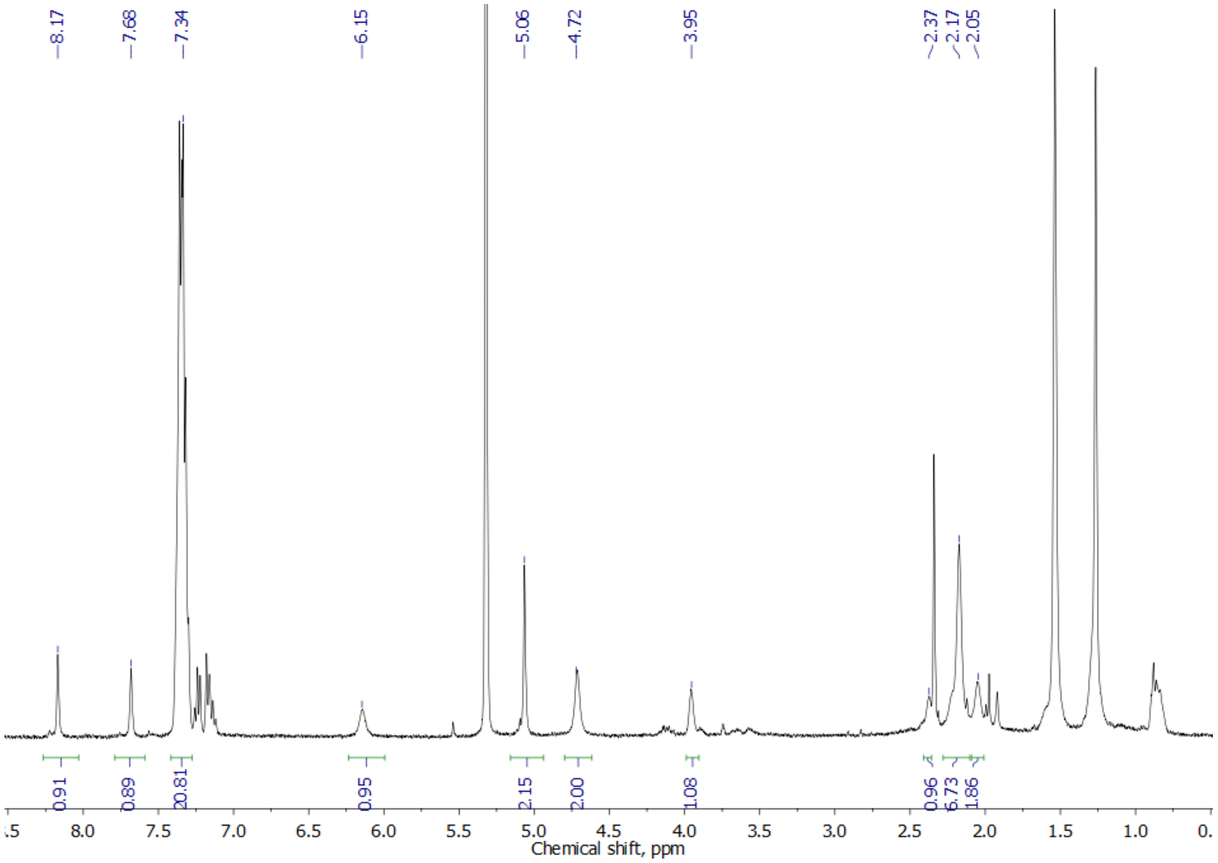

Figure S23. $^{1}H\{^{11}B\}$ NMR spectrum of the carboranoclathrochelate $FeBd_2(HGmSpCarb)(BF)_2$ (400.13 MHz, CD_2Cl_2, 20°C)

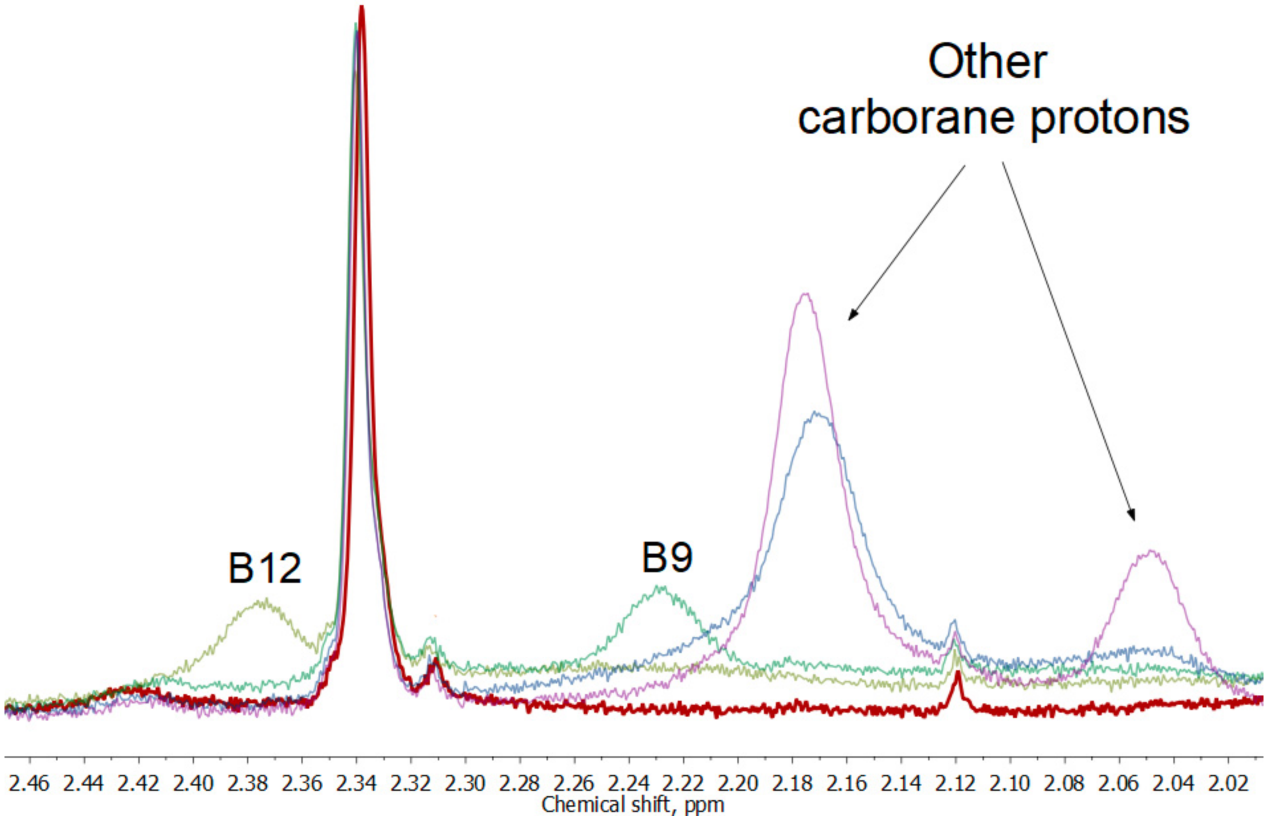

Figure S24. ^{1}H and ^{1}H selective ^{11}B-decoupled NMR spectra of the carboranoclathrochelate $FeBd_2(HGmSpCarb)(BF)_2$. The bold marron line shows its ^{1}H spectrum, other lines show the ^{1}H selective ^{11}B-decoupled spectra (yellow – $\delta(^{11}B)$ = –1.34 ppm; green – $\delta(^{11}B)$ = –4.44 ppm; blue – $\delta(^{11}B)$ = –9.39 ppm; purple – $\delta(^{11}B)$ = –11.80 ppm;).

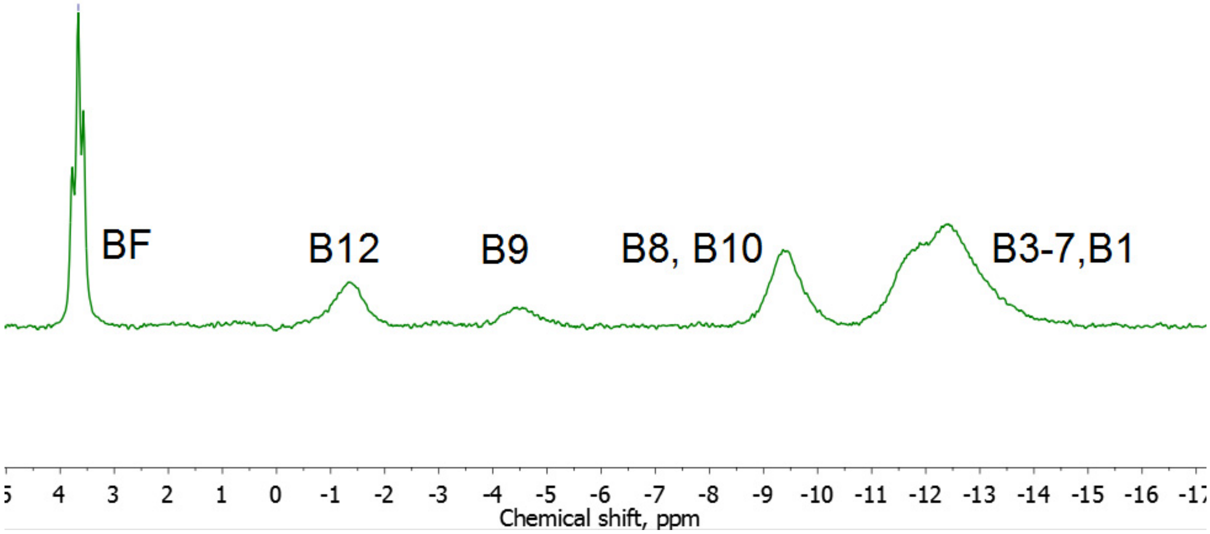

Figure S25. $^{11}B\{^{1}H\}$ NMR spectrum of the carboranoclathrochelate $FeBd_2(HGmSpCarb)(BF)_2$.

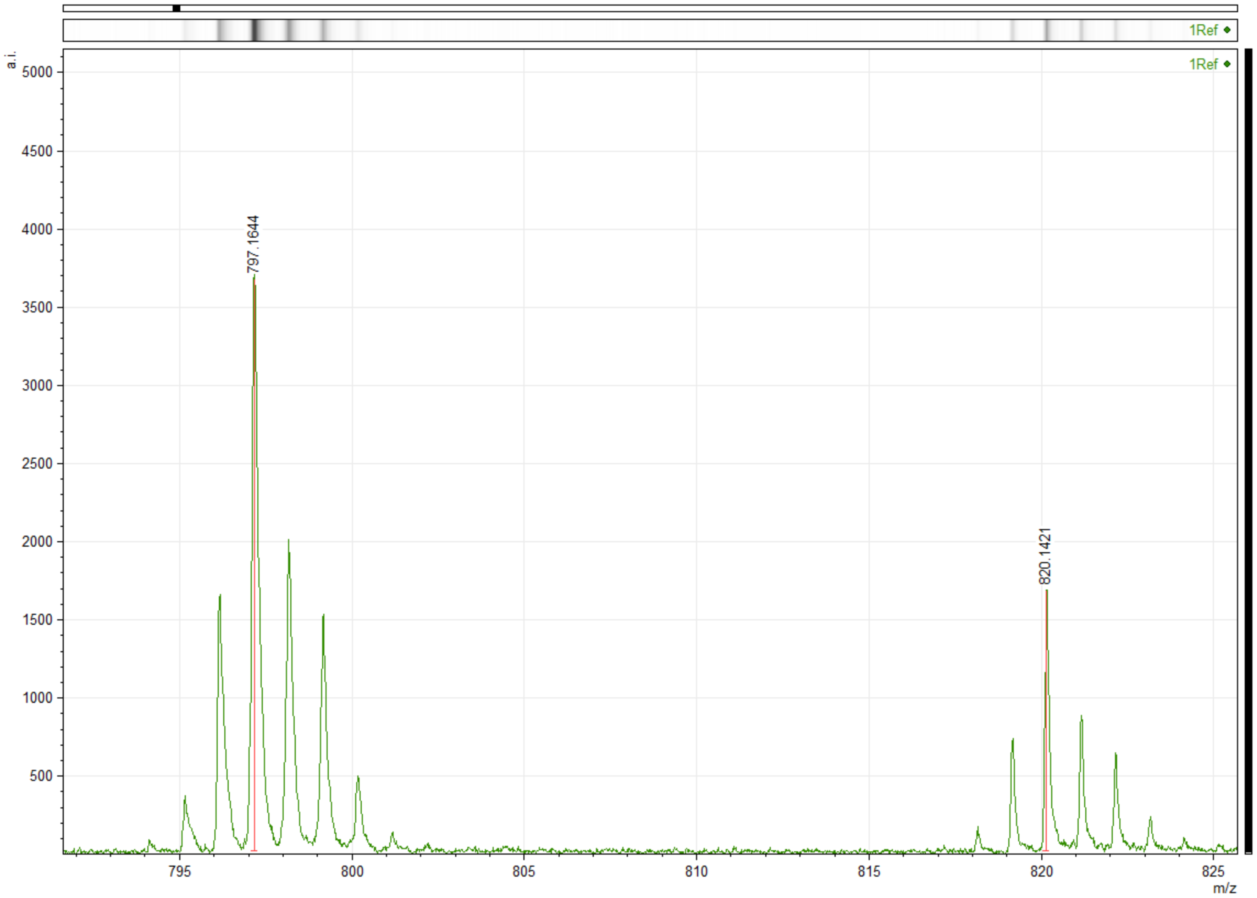

Figure S26. Fragment of MALDI-TOF spectrum of the clathrochelate precursor $FeBd_2(ClGmMorph)(BF)_2$.

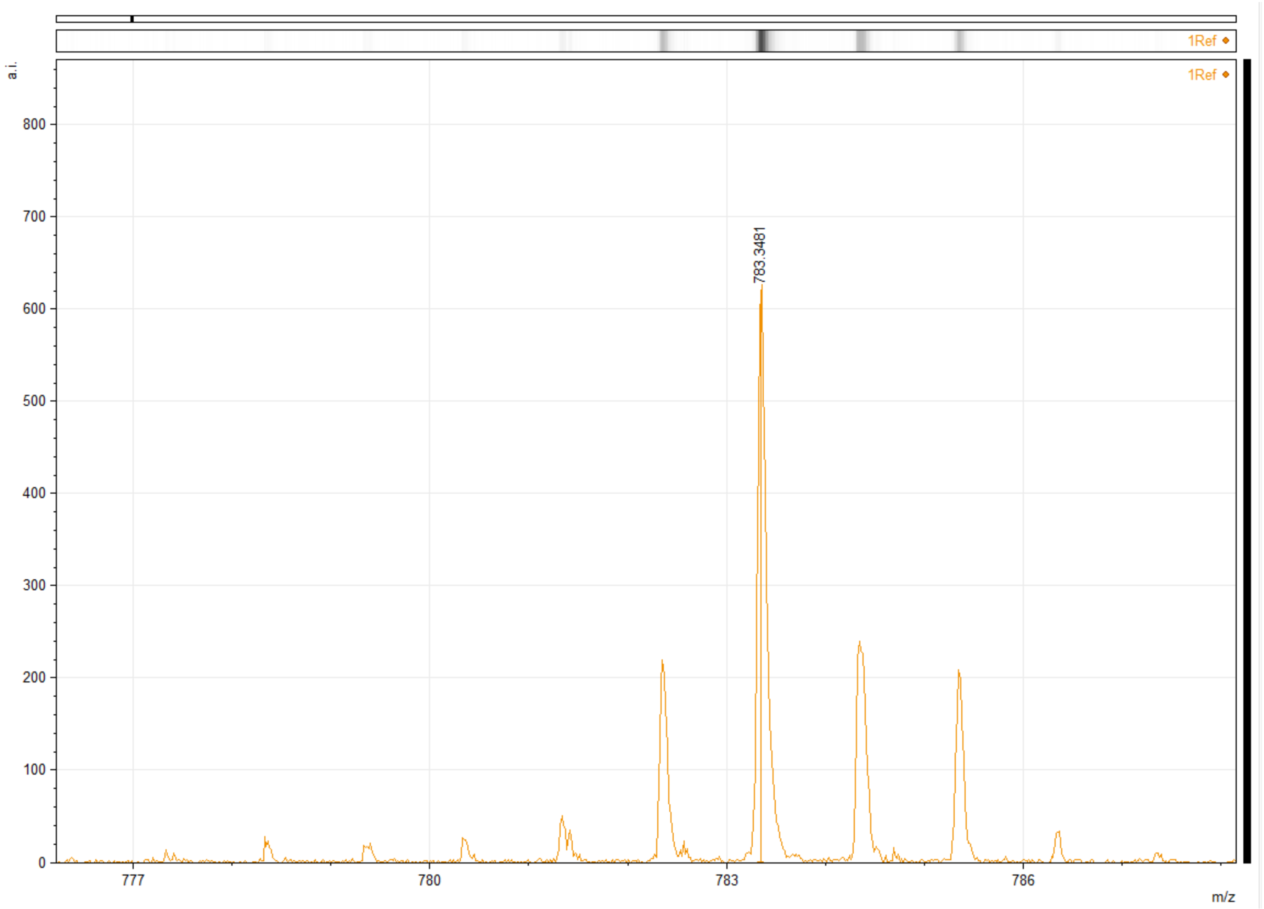

Figure S27. Fragment of MALDI-TOF spectrum of the clathrochelate precursor $FeBd_2(ClGmDea)(BF)_2$.

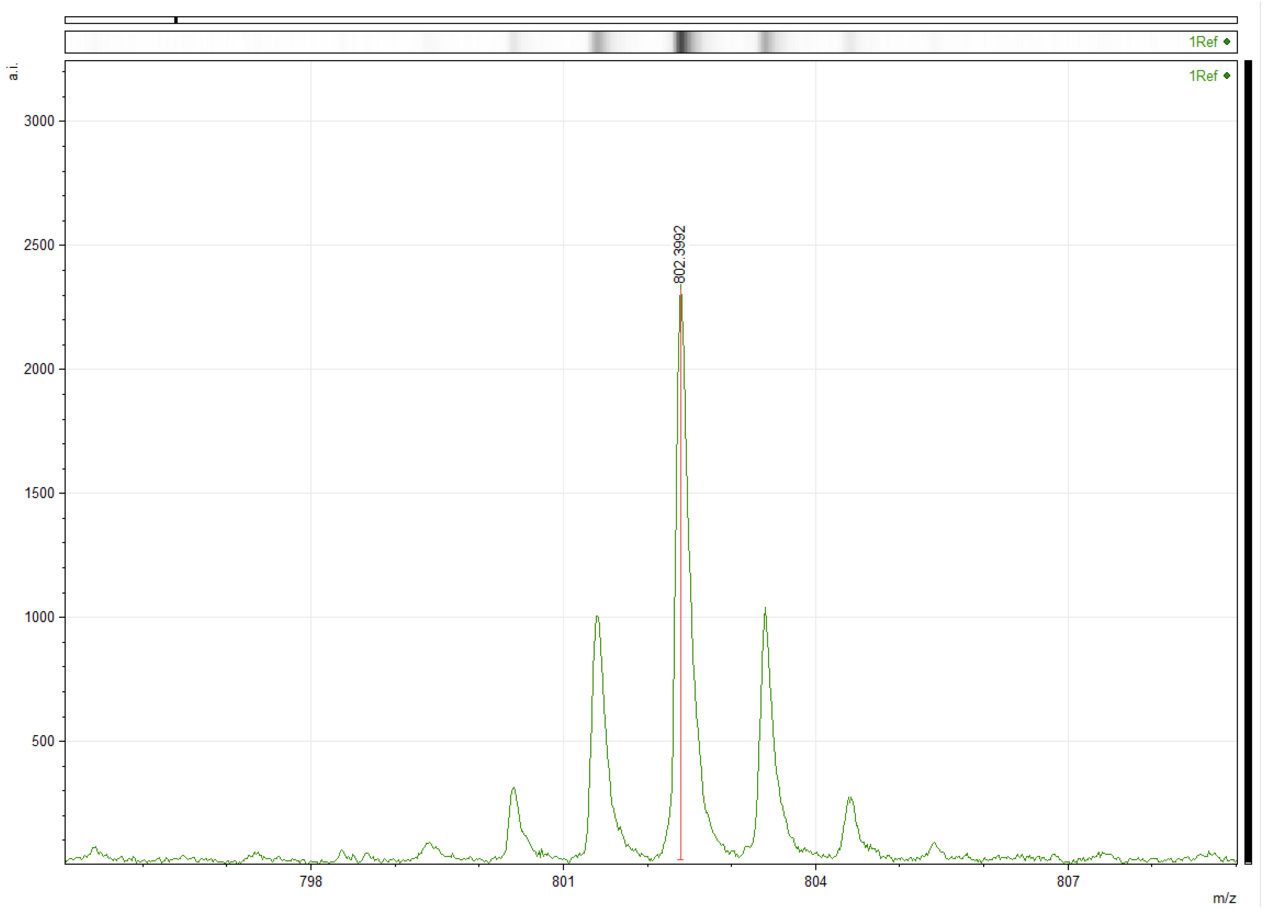

Figure S28. Fragment of MALDI-TOF spectrum of the clathrochelate $FeBd_2(PropGmDea)(BF)_2$.

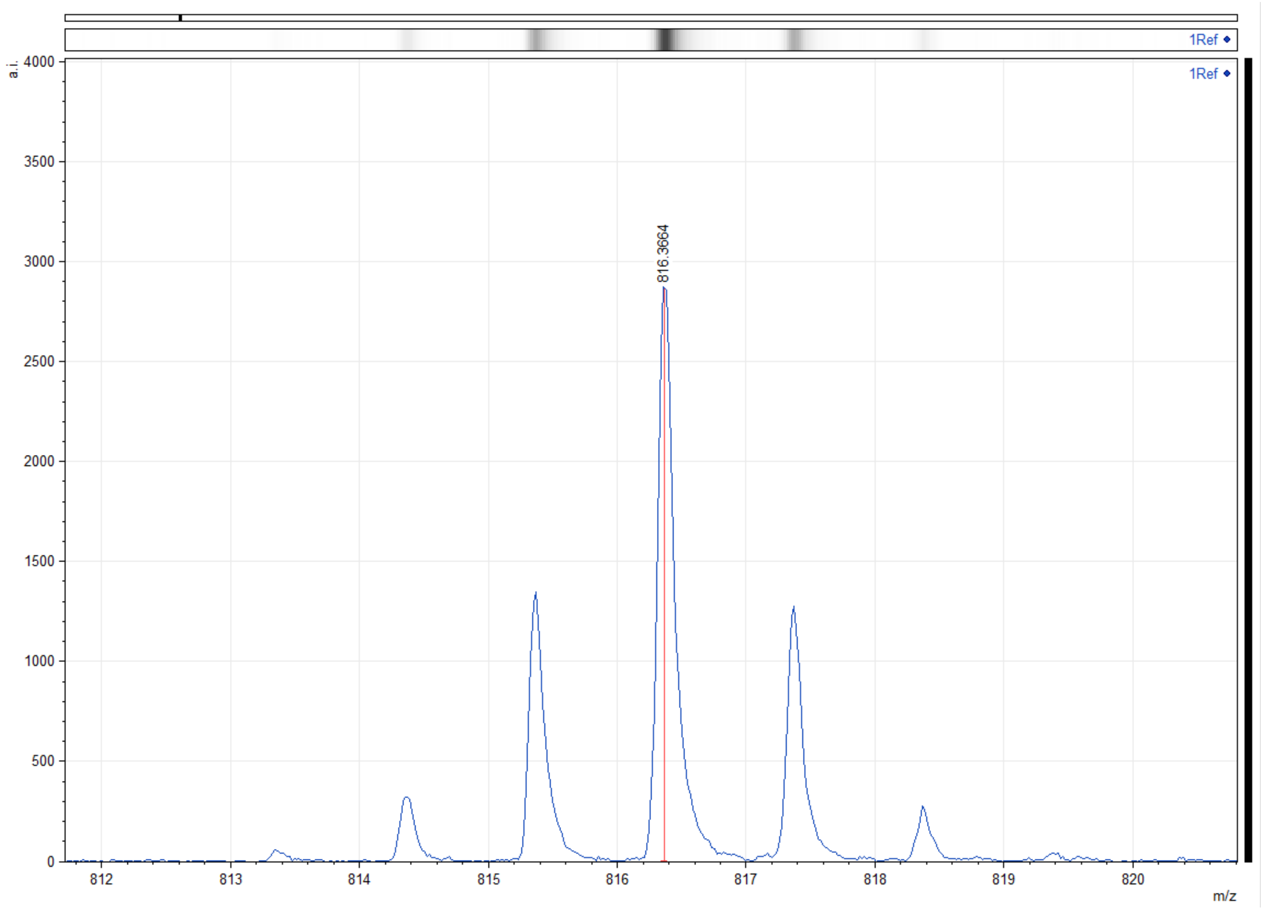

Figure S29. Fragment of MALDI-TOF spectrum of the clathrochelate $FeBd_2(PropGmMorph)(BF)_2$.

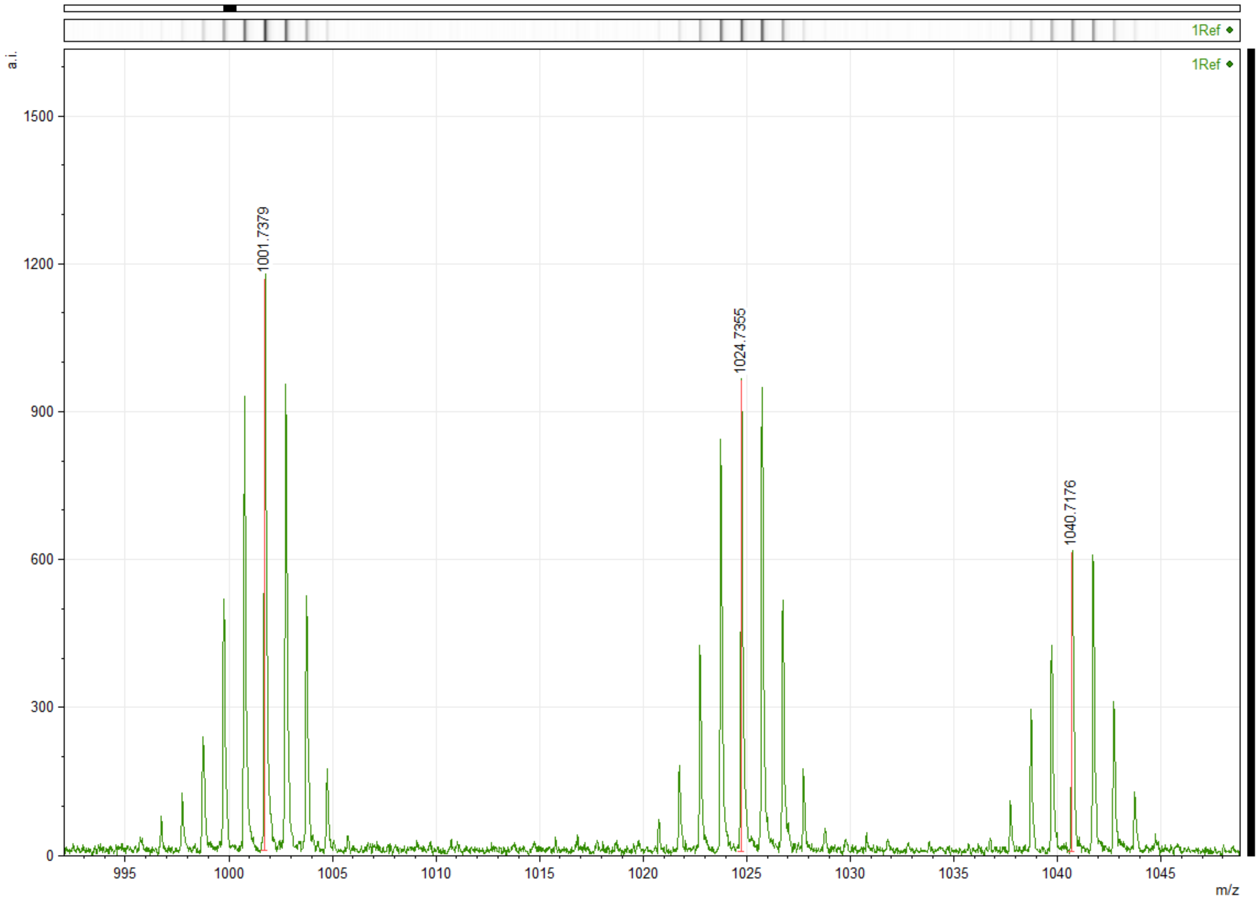

Figure S30. Fragment of MALDI-TOF spectrum of the carboranoclathrochelate $FeBd_2(DeaGmSpCarb)(BF)_2$.

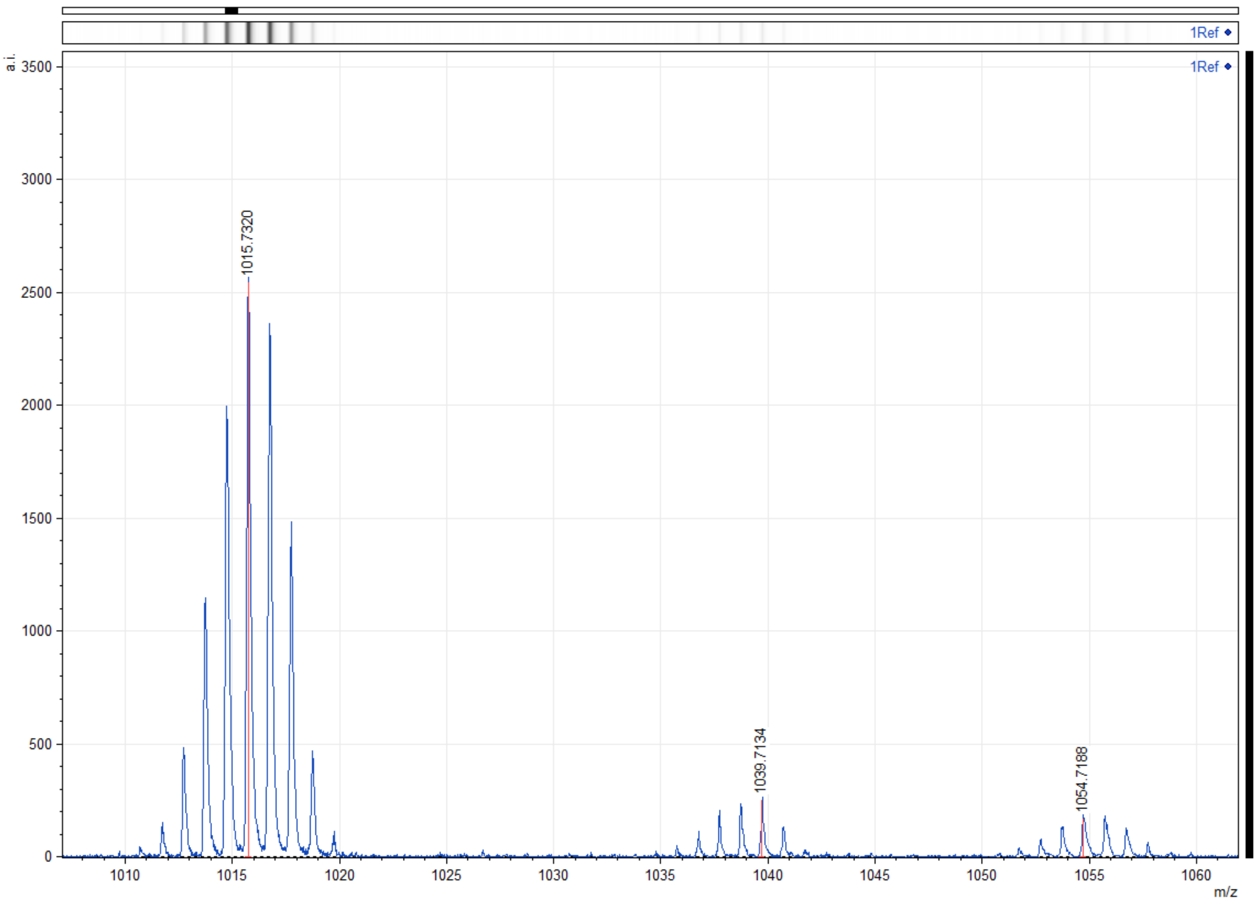

Figure S31. Fragment of MALDI-TOF spectrum of the carboranoclathrochelate $FeBd_2(MorphGmSpCarb)(BF)_2$.

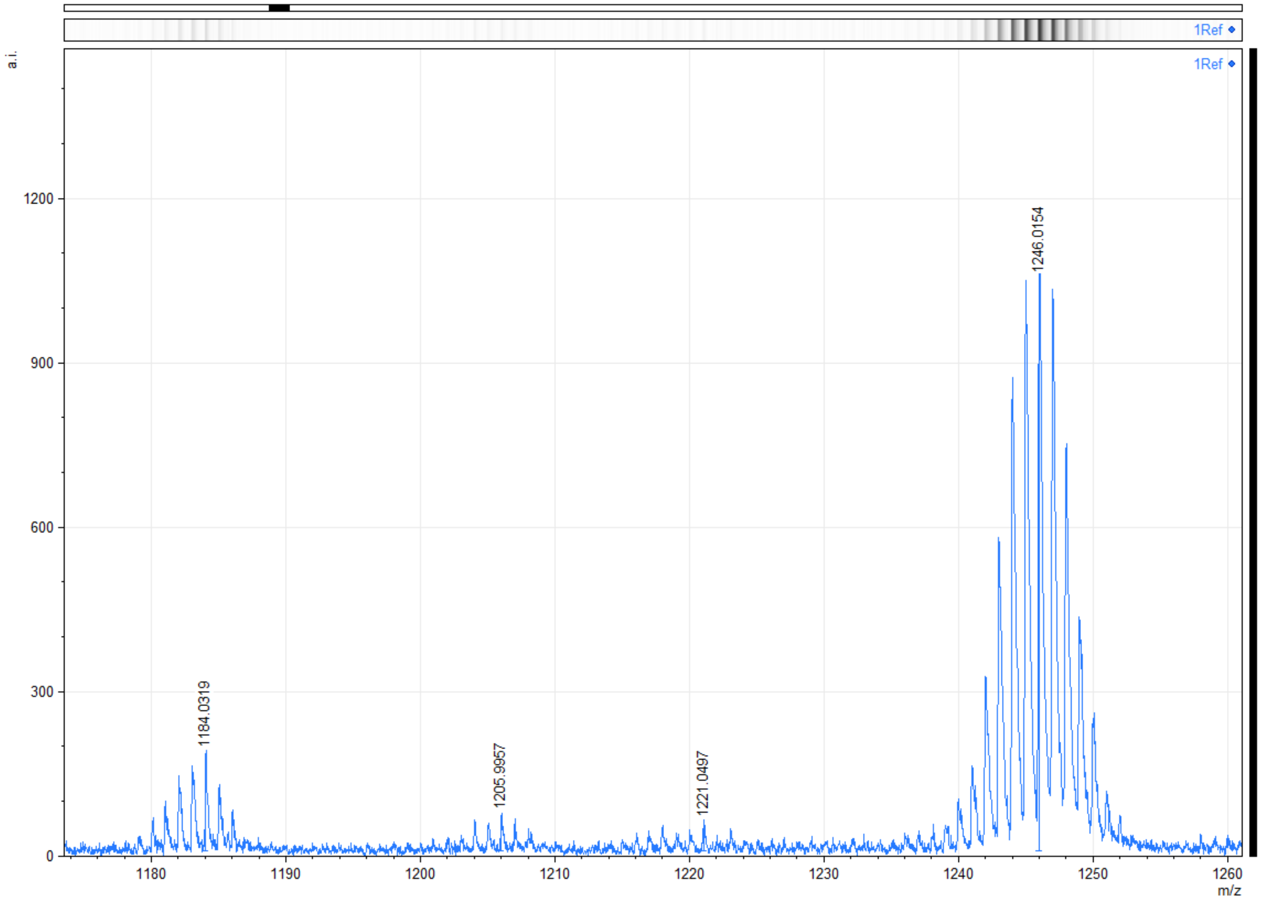

Figure S32. Fragment of MALDI-TOF spectrum of the dicarboranoclathrochelate $FeBd_2(Gm(SpCarb)_2)(BF)_2$.

Table S1. Maxima (nm) and intensities ($\varepsilon \cdot 10^{-3}$, $mol^{-1}\,l\,cm^{3}$) of the bands in the UV-vis spectra of the dichloromethane solutions of the iron(II) complexes under study and those in the spectrum of THF solution of its organic carborane-based analog

Compound	λ_1	λ_2	λ_3	λ_4	λ_5	λ_6	λ_7	λ_8	λ_9	λ_{10}	λ_{11}
$FeBd_2(Cl_2Gm)(BF)_2$	264(14)	285(7.7)	311(3.6)	399(3.2)	448(3.5)	470(19)					
$FeBd_2(ClGmMorph)(BF)_2$	240(24)	264(3.4)	279(18)	288(3.8)	342(3.1)	415(1.7)	477(17)	503(4.2)			
$FeBd_2(ClGmDea)(BF)_2$	242(31)	265(4.0)	287(1.5)	288(16)	337(3.8)	416(2.1)	481(24)	543(1.7)			
$FeBd_2(PropGmDea)(BF)_2$	243(31)	295(18)	338(2.1)	379(5.0)	434(5.1)	498(19)	525(1.8)	557(1.9)			
$FeBd_2(PropGmMorph)(BF)_2$	237(24)	251(0.6)	278(20)	298(3.8)	361(3.3)	387(22)	429(6.9)	499(21)	528(1.0)	563(1.7)	
$FeBd_2((PropNH)_2Gm)(BF)_2$	249(31)	294(16)	339(5.1)	381(1.8)	432(6.2)	491(17)	523(8.0)				
$FeBd_2(HGmProp)(BF)_2$ [25]	251(24)	278(14)	292(9.3)	335(3.3)	390(2.8)	470(24)	504(8.7)				
$FeBd_2(DeaGmSpCarb)(BF)_2$	241(29)	270(0.8)	292(14)	338(3.9)	385(0.5)	435(2.4)	489(7.0)	498(10)	524(2.9)		
$FeBd_2(MorphGmSpCarb)(BF)_2$	243(28)	270(0.5)	295(11)	313(4.1)	382(0.9)	433(1.3)	474(7.5)	501(12)	525(1.3)		
$FeBd_2(Gm(SpCarb)_2)(BF)_2$	240(24)	281(16)	365(6.5)	433(1.6)	466(1.8)	515(13)	616(1.6)				
$FeBd_2(HGmSpCarb)(BF)_2$ [25]	244(35)	265(1.0)	286(2.1)	295(3.5)	308(3.3)	336(0.5)	362(4.0)	479(24)	581(0.6)		
1-[(*o*-carboran-1'-yl)methyl]-4-pentyl-1,2,3-triazole	220(3.7)	264(0.08)									
